# Synthesis, Characterization and Use of Mesoporous Silicas of the Following Types SBA-1, SBA-2, HMM-1 and HMM-2

**DOI:** 10.3390/ma13194385

**Published:** 2020-10-01

**Authors:** Sylwia Jarmolińska, Agnieszka Feliczak-Guzik, Izabela Nowak

**Affiliations:** Faculty of Chemistry, Adam Mickiewicz University in Poznań, Uniwersytetu Poznańskiego 8, 61-614 Poznań, Poland; sylwia.jarmolinska@amu.edu.pl (S.J.); agaguzik@amu.edu.pl (A.F.-G.)

**Keywords:** mesoporous silica materials, hybrid mesoporous materials, SBA-1, SBA-2, HMM-1, HMM-2

## Abstract

Mesoporous silicas have enjoyed great interest among scientists practically from the moment of their discovery thanks to their unique attractive properties. Many types of mesoporous silicas have been described in literature, the most thoroughly MCM-41 and SBA-15 ones. The focus of this review are the methods of syntheses, characterization and use of mesoporous silicas from SBA (Santa Barbara Amorphous) and HMM (Hybrid Mesoporous Materials) groups. The first group is represented by (i) SBA-1 of three-dimensional cubic structure and Pm$\bar{3}$n symmetry and (ii) SBA-2 of three-dimensional combined hexagonal and cubic structures and P6_3_/mmc symmetry. The HMM group is represented by (i) HMM-1 of two-dimensional hexagonal structure and p6mm symmetry and (ii) HMM-2 of three-dimensional structure and P6_3_/mmc symmetry. The paper provides comprehensive information on the above-mentioned silica materials available so far, also including the data for the silicas modified with metal ions or/and organic functional groups and examples of the materials applications.

## 1. Introduction

For almost 30 years, much attention has been paid to designing and obtaining new nanomaterials. The interest in such materials stems from the fact that they have at least one component of their structure on the nanoscale (of size from 1 nm to 100 nm) and thus show a number of unique properties and have a wide range of applications [1,2]. A considerable number of such materials belong to nanoporous ones characterized by the presence of channels or pores, classified by IUPAC (International Union of Pure and Applied Chemistry) as micropores (diameter below 2 nm), mesopores (diameter in the range 2–50 nm) and macropores (diameter above 50 nm) [3,4]. The group of porous materials includes for example, zeolites [5], porous carbons [6] and mesoporous silicas [7]. The latter ones are of particular interest as they show highly ordered and stable mesoporous structure, well-developed surface area, ordered system of uniform pores of narrow size distribution and large volume, high thermal, chemical and hydrothermal stability, are nontoxic and their surface can be easily modified [1,8,9].

The history of porous materials started with discovery of natural zeolites, that are microporous aluminosilicates of crystal structure, having a developed system of micropores [10]. Their use in the chemical and petrochemical industry has brought significant benefits both to economy and the natural environment. The success of zeolites has resulted in the syntheses of a number of materials of zeolite structure, however, the size of pores was found to be a substantial limitation as they could not have been used in transformations of larger molecules [11]. The need aroused to obtain mesoporous materials, whose larger pores and large surface area could make them applicable for adsorption, separation, catalysis, as drug delivery carriers, sensors, in photonics for energy storage and conversion and as nanodevices working with large molecules [12].

The first report on the synthesis of mesoporous materials was published in the beginning of the 1990s and it has been a milestone in materials chemistry. In 1992, the first ordered mesoporous silicas were synthesized by the Mobile Research and Development Corporation [13,14]. The materials obtained were called the M41S family and included MCM type materials (Mobile Composition of Matter): MCM-41, MCM-48 and MCM-50, differing in the type of pore ordering. These silicas showed well-developed surface area and a uniform size pore system [15]. The synthesis of M41S materials has opened the way to obtaining new ordered mesoporous silicas [16]: SBA (Santa Barbara Amorphous), MSU (Michigan State University), FSM (Folded Sheet Materials), FDU (Fudan University) and KIT (Korean Advanced Institute of Science and Technology). They were synthesized by modifications of the earlier proposed method by addition of different compounds directing structural development [8,17].

Syntheses of ordered mesoporous silicas of well-defined structure need first of all precise planning of the process, the choice of a suitable compound directing structural development and a suitable precursor of silica. At a proper molar ratio of substrates and proper conditions of synthesis, such as: the time and temperature, pH of solution, time of hydrothermal treatment (ageing), the way and conditions of removal of the structure directing compound from silica pores, it is possible to obtain silicas of desired pore size and structure [18,19].

The best known and most thoroughly studied so far are the SBA-1 type silicas. In general SBA type silicas (SBA-11, SBA-12, SBA-15, SBA-16) are obtained using non-ionic surfactants as structural directing agents [20], however, SBA-1 has been for the first time synthesized using a cationic surfactant with a large head component of its molecule [21]. SBA-2 was synthesized using a gemini surfactant. Perhaps because of the necessity of independent synthesis of these two surfactants needed for obtaining SBA-1 and SBA-2, these two silicas have not been so thoroughly described in literature as the other SBA type materials synthesized with the use of commonly available non-ionic surfactants. It should be emphasized that the 3D system of pores present in SBA-1 ensures easier accessibility of these pores to the reagent molecules than the 1D cylindrical pores, so SBA-1 has high application potential, for example, in adsorption and catalysis [22].

HMM materials are organic-inorganic hybrids that have been obtained as a result of condensation of bis-silylated organic compound (R’O)_3_Si–R–Si(OR’)_3_ used as a precursor of silica. The type of material obtained, HMM-1 or HMM-2 of different structures, depends on the proportions of the reagents in the reaction mixture [19]. The materials belong to the group of mesoporous materials referred to as PMOs (Periodic Mesoporous Organosilicas).

This paper presents the information on syntheses of SBA-1, SBA-2, HMM-1 and HMM-2 silicas, their physicochemical characterization and applications, from the time they have been for the first time obtained till the present, collected on the basis of literature.

## 2. SBA-1 Silicas

### 2.1. General Characterization of SBA-1

**Figure 1:**
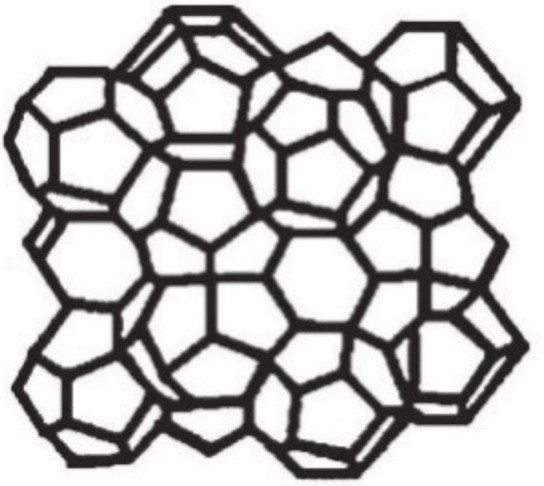
Structure of SBA-1. Reprinted from Reference [27] with permission from The Royal Society of Chemistry.

Mesoporous SBA-1 silicas have three-dimensional cubic structure of Pm$\bar{3}$n symmetry with open 3D cage type pores joined through open widows (Figure 1) [23,24]. The materials of SBA-1 type show unique textural properties, including the specific surface area in the range 1200–1450 m^2^/g and pore diameters varying from 2.1 nm to 2.6 nm. The 3D pore network is resistant to blocking and provides a large number of adsorption sites thanks to the large specific surface area. Moreover, the pores are easily accessible to the reagents molecules [25,26]. Thanks to the textural parameters and high thermal stability, SBA-1 is considered as a good catalyst support [23]. Its cubic structure is more stable than that of the silicas of hexagonal structure (MCM-41 or SBA-15). Stability of the silicas is of particular importance because the catalysts supported on them are often subjected to processes endowing them with a required type of form (e.g., tablets) [26].

Synthesis of SBA-1 material of ordered and stable structure has been a serious challenge. From the first synthesis by Huo et al. [21], many procedures for obtaining SBA-1 materials have been proposed. Below we present the procedures for the synthesis of SBA-1 materials, both unmodified and modified with metal ions or organic functional groups and their selected applications.

### 2.2. Selected Procedures for the Synthesis of SBA-1 Silicas

SBA-1 silica was for the first time obtained in 1994 by Huo et al. [21]. These authors reported that a silica mesophase of SBA-1 was formed as a result of an assembly between the cationic surfactant (C_n_H_2n+1_(C_2_H_5_)_3_N^+^, where n = 12, 14, 16, 18) with a large head group (alkyl triethylammonium group), halogen anion and cationic inorganic silicon precursor, in a strongly acidic environment. Of key importance was the use of a large surfactant molecule that favored the formation of SBA-1 structure. The new cubic mesophase of Pm$\bar{3}$n symmetry was obtained upon addition of tetraethyl orthosilicate (TEOS) to the acidic solution containing the above specified surfactant [21,28,29]. The SBA-1 silica produced in this way showed large specific surface area (1256 m^2^/g, BET) and the network of uniform size pores of 2 nm in diameter, whose presence was not manifested by a hysteresis loop in the nitrogen sorption isotherm. No presence of micropores was evidenced, so a continuous structure of pores was concluded [29]. Huo et al. have not described the SBA-1 synthesis in details, for instance they did not specify the temperature and time of the process. In 1999, Kim and Ryoo [30] examined the conditions of SBA-1 synthesis in order to obtain high quality materials and provided the details. The first stage was the synthesis of the surfactant—hexadecyltriethylammonium bromide (C_16_TEAB), which was not commercially available at that time, in the reaction of 1-bromohexadecane with the equimolar amount of triethylamine in ethanol solution. The product was carefully identified on the basis of the ^1^H NMR spectra. At the second stage, the SBA-1 synthesis was performed. The conditions of this process were adjusted to obtain the product of the best structural properties, characteristic of the SBA-1 materials. After optimization of the synthesis conditions, an excellent Pm$\bar{3}$n mesophase was obtained at the molar ratios of the reagents of: 1C_16_TEAB:5TEOS:280HCl:3500H_2_O. The surfactant, acid and water were stirred until a homogeneous solution, then the mixture was cooled to 0 °C in an ice bath. Then TEOS cooled to the same temperature was added to the surfactant solution upon stirring. The stirring was continued for 4 h, that is till completion of precipitate formation, then within 10 min the mixture was heated to 100 °C, maintained for 1 h. The precipitate was filtered off without washing, dried under vacuum at room temperature and then heated for 30 min at 60 °C in a drier. The product was washed with a mixture of HCl/ethanol, then dried again at 60 °C for 30 min and finally calcinated at 550 °C. XRD (X-ray diffraction) and MAS ^29^Si NMR studies have shown that heating of the product of synthesis for 1 h at 100 °C results in a significant improvement in the silica cross-linking preserving its cubic mesostructure. A high-quality diffractogram of SBA-1 showed three well-resolved reflections corresponding to the lattice planes (200), (210) and (211) in the Bragg angle range 2°–3° [30]. In 2000, Sakamoto et al. [24] obtained SBA-1 synthesized in acidic conditions at 0 °C. The product of their synthesis had a 3D cubic structure of Pm$\bar{3}$n symmetry with uniform cage type pores with open windows. The small-angle diffractogram of this SBA-1 revealed two well-resolved reflections in the 2θ range 2°–3° along with the well-resolved reflections at the angles corresponding to the planes: (200), (210) and (211) [24].

To get more information on the porous structure of SBA-1 ordered silica, Kruk et al. [31] have applied the method of nitrogen adsorption in a wide range of relative pressures. They synthesized SBA-1 using C_16_TEAB as a surfactant and TEOS as a silicon precursor, at 0 °C. It was important not to wash the obtained precipitate with distilled water or organic solvents as then the silica structure would be lost. In order to obtain highly ordered structure, the preparation was not washed after the synthesis. The dried product of synthesis was washed with a mixture of HCl/ethanol/water and then calcined at 550 °C. The obtained SBA-1 showed very good textural properties: specific surface area of 1180 m^2^/g, mean pore diameter of 3 nm and total pore volume of 0.69 cm^3^/g. However, the pore size distribution was relatively wide, so the material obtained must have contained also smaller size diameter. SBA-1 materials contain two types of pores, so the results obtained by the BJH (Barrett- Joyner-Halenda) method were unreliable as they were produced assuming the presence of cylindrical pores only. The BJH results suggested that the change in the pore diameter was a result of the presence of cage pores [31].

Che et al. [32] have also proposed a method for the synthesis of highly-ordered SBA-1 of well-defined crystal morphology with a large number of facets, which has been confirmed by SEM (Scanning Electron Microscopy) image analysis. The reaction mixture they used contained 0.13 C_16_TEAB:1TEOS:2.5HCl:125H_2_O stirred for 4 days at temperatures from −5 °C to 40 °C. The XRD diffractogram showed a broad reflection at 2θ close to 20°, which indicated that the walls of the material obtained were amorphous. The small-angle diffractograms of the synthesis products obtained at −5 °C, 0 °C or 5 °C revealed the presence of three well-resolved reflections for Bragg angles from 1.5° to 3.0°, characteristic of materials of Pm$\bar{3}$n symmetry. With increasing temperature of synthesis, the intensities of the reflections decreased. The use of low temperatures of synthesis was conducive to formation of highly-ordered cubic mesophase of SBA-1 [32]. Another stage of the study was optimization of the conditions of synthesis of SBA-1 material modified with molybdenum ions [23]. These authors considered two aspects: the molar ratio of H_2_O/HCl and Si/Mo. Che et al. have proved that pH of the reaction mixture was the most important parameter of SBA-1 formation and incorporation of molybdenum to the structure of this silica. In the presence of Mo ions the cubic mesophase formation was the easiest for the molar ratio of H_2_O/HCl of 50. It may be related to the fact that the concentration of hydrogen ions (H^+^) has a strong impact on silicates condensation. At the optimized pH of the reaction mixture, Che et al. obtained a highly-ordered SBA-1 structure with Si/Mo molar ratio of at most 23 [23].

#### 2.2.1. The Use Surfactants Other Than C_16_TEAB in SBA-1 Synthesis

**Figure 2:**
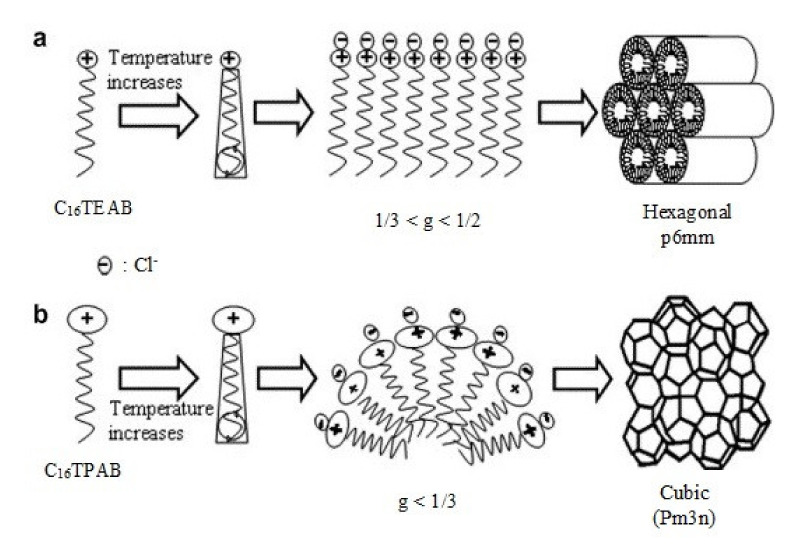
Schemes of changes in the ordering of surfactant micelles obtained with increasing temperature for (**a**) C_16_TEAB and (**b**) C_16_TPAB. Reprinted with permission from Reference [33] with minor modification.

The choice of surfactant for the synthesis of SBA-1 is of great significance. The main parameter characterizing surfactants is its packing [22,33], defined as:

(1) $$g=\frac{V}{a_{0}l}$$

where *V* stands for the total volume of the surfactant chain, *a_0_* is the area of the surfactant head and *l* is the length of the surfactant chain. This parameter also characterizes the geometry of the mesophase products. The structural properties of surfactant, including the number of carbon atoms in the hydrophobic chain, degree of chain saturation and the charge of surfactant molecule head, to a significant degree determine the direction of ordering of the forming mesophase and its final structure. The surfactant whose molecules have large heads are characterized by a smaller packing parameter *g*, which permits formation of conical shapes of a surfactant molecule. Then its molecules can be effectively organized into spherical micellar structures. As the structure of SBA-1 corresponds to the micellar structure of high surface curvature (*g* < 1/3), the size of the surfactant molecule head is an important factor controlling the formation of this material. Small values of *g* correspond to a stable structure of greater curvature, (e.g., cubic SBA-1 of *g* < 1/3), while high *g* values correspond to a stable structure of smaller curvature (e.g., hexagonal SBA-3 of 1/3 < *g* < 1/2). Kao et al. [33] have been the first to use hexadecyltripropylammonium bromide (C_16_TPAB) whose molecules have heads even larger than that of C_16_TEAB. The synthesis was carried out in a wide range of temperatures, from 0 °C to 90 °C, in a strongly acidic environment controlled by addition of HCl. Kao et al. have explained the role of surfactants C_16_TPAB and C_16_TEAB in the formation of cubic mesophase. At low temperatures of synthesis with C_16_TEAB its *g* < 1/3, so this surfactant can be used for the synthesis of SBA-1 in low temperatures. With increasing temperature, the conformation of the surfactant hydrocarbon chain is disturbed leading to an increase in its effective volume so also to an increase in *g*. Thus, the micellar structure of C_16_TEAB is not stable above 50 °C, which leads to formation of hexagonal phase. The use of C_16_TPAB whose molecules heads have larger area (*a_0_*), which means that the chain volume *V* increases, eliminates the phase transition with increasing temperature. The close packing of C_16_TPAB molecules means that the spherical micelles are more resistant to temperature changes, so this surfactant can be used for the synthesis of SBA-1 at higher temperatures [33]. Figure 2 presents the phase transitions of the surfactant at high temperatures when using C_16_TEAB or C_16_TPAB surfactants [33].

In view of the fact that C_16_TPAB is not commercially available, attempts have been made to use some easily accessible surfactants for the SBA-1 synthesis. Che et al. [34] have used hexadecyltrimethylammonium bromide (C_16_TAB), so the surfactant used for the synthesis of MCM-41 and SBA-3. The cubic mesophase of Pm$\bar{3}$n symmetry was obtained taking the reagents at the following molar ratios: C_16_TAB:TEOS:HCl:H_2_O of 0.2:1:27:125. When the reaction temperature was reduced to 0 °C, the time of TEOS condensation was 80 min. The XRD pattern of the SBA-1 material obtained showed three well-resolved reflections in 2θ from 1.5° to 3°, corresponding to the planes (200), (210) and (211) but also additional weak reflections in the range 3.5°–6° evidencing the presence of the planes (220), (310), (222), (320), (400), (420) and (421), which indicated a high degree of ordering of the silica structure obtained. A decrease in the molar ratio of H_2_O/HCl in the reaction mixture resulted in a lowering of the degree of ordering in the final SBA-1 material, while the use of a higher molar ratio of H_2_O/HCl led to a mixture of SBA-1 and SBA-3 phases which with time converted fully to the hexagonal structure of SBA-3 [34].

#### 2.2.2. Precursors of Silicon Applied in the Synthesis of SBA-1

A new procedure of SBA-1 synthesis was proposed by Chao et al. in 2003 [35]. They observed changes in the SBA-1 crystal morphology in response to changes in pH of the reaction environment in the range 1.0–2.0, close to the isoelectric point of silica (IEP). As the rate of silica condensation depends on pH, with pH increasing from 1 to 2, the SBA-1 crystals morphology changed from sphere (pH = 1), through octadecahedron (pH = 1.5), truncated cube (pH = 1.8) to cube (pH = 2) [35]. These observations imply that in order to obtain crystals of well-developed morphology, the rate of their growth must be low. On the basis of the chemistry of silica, near its IEP, at pH close to 2.0, the rate of silica condensation is the lowest and its surface charge is neutral. That is why in the standard synthesis of SBA-1 in acidic environment, TEOS is commonly used as a precursor of silicon. Chao et al. have been the first to use diluted acidic solution of sodium silicate of pH from 1.0 to 2.0 and a surfactant C_18_TMACl (octadecyltrimethylammonium chloride). Thus, the self-organizing mesostructure of ternary cationic ammonium surfactant and silica may form slowly because of weak hydrogen bonds and slow condensation of silica [35]. The use of alkyltrimethylammonium surfactants of a smaller number carbon atoms in the hydrocarbon chain (n = 14 or 16) in the same conditions of synthesis have also led to materials of desired cubic structure of Pm$\bar{3}$n symmetry [36].

As follows from the above examples, the role of silica precursors in the synthesis of SBA-1 in strongly acidic conditions is important. Of decisive impact on the character and quality of the final mesosphere is the type of alcohol obtained as a result of hydrolysis of the alkoxysilane used. In order to prevent the formation of undesirable mesophase in the conventional process of SBA-1 synthesis at high temperatures, tetramethyl orthosilicate (TMOS) can be used as a silicon precursor instead of tetraethyl orthosilicate (TEOS). The relevant studies have been performed by Kao and Cheng [37], who proved that when using TMOS, the structure of SBA-1 is preserved even in the process at 70 °C, while with TEOS already at 50 °C the structure of the silica was disturbed. The use of TMOS molecules instead of TEOS as a source of silica enables the formation of an orderly mesostructure SBA-1 during the synthesis at different temperatures. It indicates that the methanol molecules formed by TMOS in the process of hydrolysis can act as a better controller of the phase formation than the ethanol molecules released from TEOS, in order to maintain cubic mesostructure at higher temperatures [37].

**Figure 3:**
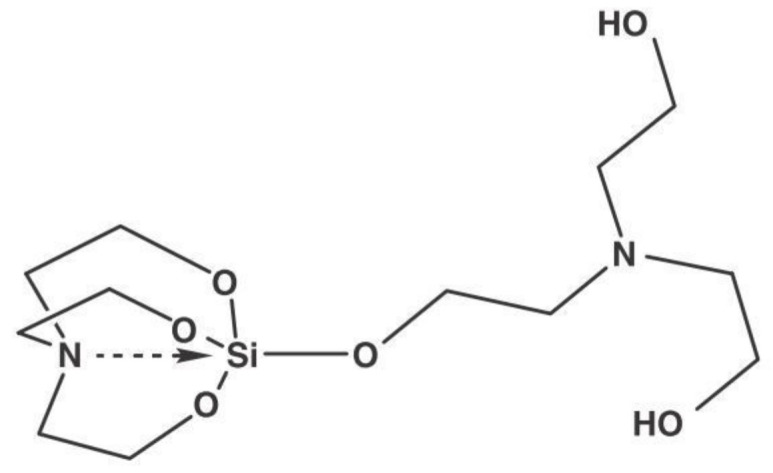
Structure of silatrane. Reprinted with permission from Reference [38]. Copyright (2007) The American Ceramic Society.

Tanglumlert et al. [38] have described the effects of using silatrane in the SBA-1 synthesis as a precursor of silicon. Silatranes are a very interesting group of organosilicon compounds, showing a specific structure and wide application potential (e.g., Figure 3). They show cage type structure in which silicon atom is additionally coordinated with nitrogen [39]. These compounds have been successfully used as a source of silicon in the syntheses of microporous zeolites [40] and mesoporous materials [41]. Tanglumlert et al. have synthesized silatrane with the use of fumed silica SiO_2_ and triethanolamine. For the SBA-1 synthesis they used trimethylammonium surfactants with a small head (C_n_TAB, with n = 14, 16, 18). Thanks to this choice they were able to check the effects of the alkyl chain length, pH reaction mixture and synthesis temperature on the degree of ordering of the material. Their results have proved that the use of silatrane permits obtaining well-ordered and stable mesoporous SBA-1. Moreover, these authors have shown that triethanolamine molecules obtained with the use of the silicon precursor had a significant impact on the structure of micelles of the surfactants through reduction in the packing parameter (*g*) [38]. The structure of silatrane is shown in Figure 3.

Knowing that using silatrane as the source of silicon, a stable and well-ordered structure of unmodified SBA-1 material was obtained, Tanglumlert et al. have also used silatrane for the synthesis of SBA-1 modified with iron or titanium ions by the method of co-precipitation, at room temperature and with C_16_TAB [42]. Also in these syntheses silatrane proved to be a highly reactive precursor for obtaining well-ordered SBA-1 modified with transition metal ions. SBA-1 containing up to 6% wt. of iron ions or up to 10% wt. of titanium ions were characterized by stable and ordered structures, in which part of Si^4+^ ions had been replaced with Fe^3+^ or Ti^4+^ ones [42]. In their further studies, Tanglumlert et al. [43] have used the catalysts obtained in the reaction of styrene oxidation with H_2_O_2_ in which the catalysts were active and selective. However, the highest activity showed the catalysts containing 4% wt. of Fe or 2% wt. of Ti ions. Moreover, as follows from a comparison of the catalysts obtained by co-precipitation and impregnation methods, the use of the ones obtained by co-precipitation ensured higher degrees of styrene conversion [43].

#### 2.2.3. The Reaction Environment and Stability of SBA-1 Materials

Synthesis of SBA-1 materials of highly ordered structure of Pm$\bar{3}$n symmetry needs a strongly acidic environment. The type of acid used for pH regulation was of key importance in SBA-1 synthesis. Most often hydrochloric acid has been used for the synthesis of SBA-1 materials as its presence in a proper concentration favored the formation of the cubic Pm$\bar{3}$n structure. Kao et al. [33] have tested the effects of HCl and HBr on the structure of the synthesized SBA-1 materials. According to their results, the synthesis with the use of HBr did not lead to a cubic structure. The impact of halogen ions on formation of mesoporous structures can be explained by the strength of their adsorption on the heads of the surfactant molecules making micellar structures. The mesophase of SBA-1 is formed until the surfactant micelles develop into spherical shape. Adsorption of halogen anions increases the effective area of the surfactant molecules heads, which significantly contributes to increase in the curvature radius of the micelles. The halogen anions in a surfactant solution are more or less hydrated. The poorer hydrated ions have smaller ionic radii and thus stronger bind to the surfactant head. The ionic radius of Cl^−^ is greater than that of Br^−^. Smaller anions reduce the electrostatic repulsion between the heads of cationic surfactants, which leads to decrease in the effective area of surfactant. Thus the use of HBr leads to a higher *g* parameter, so the formation of hexagonal SBA-3 (1/3 < *g* <1/2) is preferred. In order to maintain the value of *g* smaller than 1/3, to obtain a cubic SBA-1, the choice of acid is of vital importance [33].

Vinu et al. [44] have studied the effect of HCl/surfactant molar ratio and the time of ageing at 100 °C on the structure of SBA-1. According to these authors, the samples obtained at the molar ratio of HCl/surfactant of 280 showed the structure of the highest degree of ordering, higher specific surface area and higher total pore volume than the samples synthesized at lower HCl/surfactant ratios. As to the effect of the time of ageing, its extension from 1 h to 72 h caused an increase in the mean pore diameter from 2.3 nm to 3.0 nm and increase in the total pore volume from 0.70 cm^3^/g to 1.03 cm^3^/g but a decrease in the specific surface area from 1430 m^2^/g to 1100 m^2^/g. Moreover, the extension of ageing time caused an increase in the SBA-1 resistance to contact with water, which was highly desirable as in other conditions the precipitates could not have been washed with water for the risk of the product destabilization. Low resistance of SBA-1 to water is a consequence of a low degree of silica walls cross-linking, which makes them susceptible to hydrolysis thus leading to structural damage. Vinu et al. have shown that the resistance of SBA-1 towards water increases with increasing time of crystallization. This increase in stability is probably a result of enhanced cross-linking of silica, which hinders the hydrolysis. The same authors examined the mechanical strength of SBA-1, which proved to be higher than those of MCM-41 and SBA-15 but comparable to that of MCM-48 [44].

#### 2.2.4. The Auxiliary Compounds Stabilizing the Structure of SBA-1

The resistance of SBA-1 to contact with water can also be enhanced by addition of auxiliary compounds. Kao et al. [45] have applied D-fructose as an auxiliary compound in the synthesis of SBA-1 and established that its presence increased the SBA-1 resistance to water. The same authors have been the first to publish a successful synthesis of SBA-1 at temperatures higher than 40 °C, with absolutely no phase transformation. In the procedure they described, D-fructose was mixed with HCl and water at room temperature, then TEOS was added followed by C_16_TEAB. The mixture was cooled to a minimum temperature of 0 °C or heated to a desired temperature, maximum of 90 °C, upon vigorous stirring. Thanks to the use of D-fructose, which is a cheap and environmentally friendly compound, the obtained SBA-1 materials were highly-ordered and stable in a wide range of temperatures. The synthesis proposed by these authors has shown the possibility of obtaining SBA-1 modified with organic groups by direct synthesis [45].

**Figure 4:**
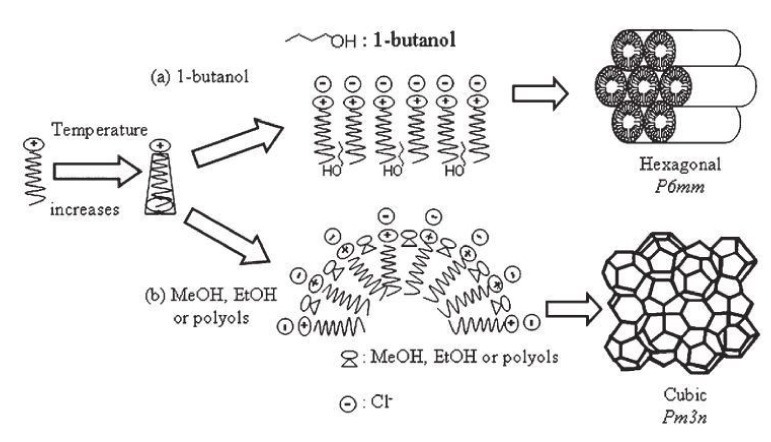
The proposed schema of changes in organization of a surfactant with increasing temperature in the presence of (**a**) 1-butanol and (**b**) ethanol, methanol or polyols. Reprinted from Reference [27] with permission from The Royal Society of Chemistry.

Similarly effective in maintaining cubic structure of SBA-1 in a wide range of temperatures is the addition of short-chain alcohols, for example, methanol or ethanol or polyols such as glycerol or D-sorbitol, which has been proved by Kao et al. [27]. A higher temperature of synthesis causes an increase in the degree of silica cross-linking leading to materials of increased stability and it is conducive to faster formation of the cubic mesostructure of SBA-1. A schema of changes in the organization of a surfactant with increasing temperature, in the presence of different alcohols is shown in Figure 4.

As can be seen, the use alcohols containing four carbon atoms, for example, 1-butanol, does not promote the formation of the desired regular structure—a material with hexagonal structure is formed. On the other hand, the addition of short-chain alcohols (more hydrophilic ones) during the synthesis at higher temperatures leads to the desired cubic structure (SBA-1 material). The placement of alcohol molecules (which acts as a solvent) in the outer boundaries of the surfactant heads causes the enlargement of the effective area of the surfactant molecules heads (*a_0_*), which balances the effect of increased volume caused by temperature increase and thus maintains the *g* value in favor of the formation of the cubic phase SBA-1 [27].

#### 2.2.5. Complex of Polyelectrolyte-Surfactant as a Template in Acidic Conditions

A new procedure of SBA-1 synthesis in acidic conditions has been proposed by Pantazis and Pomonis [46]. Their conception included the use of a polyelectrolyte such as a poly(acrylic acid) (PAA) in complex with a surfactant C_n_TAB. According to the procedure they proposed, at first poly(acrylic acid) of molar mass 2000 g/mol, denoted as Pac2, was dissolved in water. The pH value of the mixture was 3.2, however, it had to be decreased to 1.5 in order to avoid complexing the polyelectrolyte with the surfactant. Subsequently TEOS was added so that the molar composition of the mixture was: 1 TEOS: 0.32 C_n_TAB: 0.01 Pac2: 253 H_2_O. Then, NH_3_ was added dropwise for 2 h in order to establish the effect of pH on the SBA-1 structure formation. The appearance of precipitate was observed at pH from the range 3.2–3.3, however, the mixture pH was brought to 4.0–7.0 as in this range the organic complex was formed and it was stirred for 24 h. Then the precipitate was filtered off, washed with water and dried at 90 °C. The final product was obtained as a result of calcination at 600 °C for 6 h. Most probably complex formation between the surfactant and carboxyl groups with increasing pH led to generation of complex chains that formed spherical micelles needed for obtaining cubic mesophase of Pm$\bar{3}$n symmetry. The used poly(acrylic acid) was probably able to stabilize the micelles in a wide range of pH. When C_16_TAB was used, the final silica showed hexagonal structure, while with C_14_TAB the product showed a desired cubic structure, characteristic of SBA-1. Moreover, depending on the molar mass of the poly(acrylic acid) and pH of the mixture, the structure of the final product changed. Thus, the use of poly(acrylic acid) of appropriate parameters permitted control of the unit cell parameters [46].

#### 2.2.6. Modification of SBA-1 With Metal Ions in Acidic Conditions and the Use of Modified Silicas in Catalytic Reactions

SBA-1 silicas with a network of three-dimensional channels are much preferred for catalytic applications than the materials with one-dimensional channels [32]. In this aspect, the synthesis of SBA-1 silicas modified with transition metal ions is of great interest. SBA-1 synthesized in acidic environment is convenient for modification with transition metal ions as many metal precursors dissolve at low pH. Unfortunately, even though the ratio of metal ions to silicon atoms in the reaction mixture is high, a small number of metal ions are incorporated into the silica upon silica condensation because of a high solubility of the metal precursor [47]. Besides, a higher concentration of the precursor can lead to formation of hexagonal mesophase instead of cubic one. Addition of increasing amounts a water solution of VCl_4_ to a standard synthetic mixture of SBA-1 resulted in the final product of hexagonal structure—SBA-3. Most probably the addition of VCl_4_ increased the concentration of chloride ions, which reduced the repulsion of the cationic surfactant heads, thus the packing of the surfactant heads increased which favored formation of hexagonal phase [48].

In view of the above, the choice of metal precursor is of particular importance. For instance, let us consider the use of two precursors of molybdenum ions: MoCl_5_ and (NH_4_)_6_Mo_7_O_24_·4H_2_O, at the same ratio of metal ions to silicon atoms. Using the first precursor Morey et al. [47] succeeded in introducing only 0.4% at. Mo ions into the SBA-1 structure because of high solubility of Mo^5+^ in a strongly acidic environment, while Dai et al. [49] who used (NH_4_)_6_Mo_7_O_24_·4H_2_O, introduced 8.7% wt. of molybdenum ions to the silica structure as the anionic species Mo_7_O_24_^6−^ favored incorporation of the metal ions into SBA-1 structure. Similarly, introduction of vanadium ions to SBA-1 was effective if ammonium metavanadate (NH_4_VO_3_) was used as their precursor [50].

Dai et al. [49] have applied SBA-1 modified with molybdenum ions introduced on the surface or into its structure by co-precipitation or impregnation for partial oxidation of methane with oxygen. The catalyst Mo-SBA-1 was obtained in acidic environment using C_16_TEAB as a surfactant, TEOS as a precursor of silicon and (NH_4_)_6_Mo_7_O_24_·4H_2_O as a precursor of molybdenum ions. The temperature of synthesis was 0 °C. Prior to addition to the reaction mixture, TEOS and (NH_4_)_6_Mo_7_O_24_·4H_2_O were also cooled to the same temperature. The reagents were stirred for 6 h. The modification by impregnation with (NH_4_)_6_Mo_7_O_24_·4H_2_O was performed with pure SBA-1 synthesized as above, without addition of metal precursor in the process. Results of XRD measurements in a small-angle range proved that the highly-ordered cubic structure was maintained for the materials containing up to 8.8% wt. of molybdenum ions introduced by co-precipitation method. These materials also showed large specific surface area of more than 1000 m^2^/g. The modified SBA-1 materials obtained by impregnation contained 9% wt. of molybdenum ions and showed the specific surface area of 718 m^2^/g, which can be explained by the loss of ordering of the crystalline structure, as evidenced by XRD patterns [49]. According to the catalytic tests, in partial oxidation of methane with oxygen, the SBA-1 modified with molybdenum ions by co-precipitation showed higher catalytic activity than pure SBA-1 and SBA-1 modified with Mo ions by impregnation. The catalysts selectivity and the yield of formaldehyde formation increased linearly with increasing content of molybdenum ions, at a similar degree of methane conversion. Thus the catalysts selectivity depended on the content and dispersion of the metal ions in SBA-1 structure [49].

Hartmann et al. [51] have tested catalytic properties of SBA-1 modified with aluminum ions introduced by co-precipitation in acidic conditions, using aluminum isopropoxide Al[OCH(CH_3_)_2_]_3_ as a precursor of aluminum species, so that the catalyst contained Brønsted acidic sites. The same authors have reported synthesis of bifunctional catalyst based on SBA-1 containing aluminum and platinum ions in order to apply it for isomerization of *n*-decane. As the precursor of platinum they used Pt(NH_3_)_4_Cl_2_ which was introduced into the water solution of SBA-1 modified with aluminum ions, then the excess of water was evaporated on a rotary evaporator. The obtained product was subjected to reduction with hydrogen at 310 °C for 4 h. The catalyst obtained showed a high catalytic activity, much higher than that of SBA-1 modified only with platinum ions [51].

SBA-1 samples modified with aluminum have been also synthesized by Peng et al. [52] who used them as heterogeneous catalysts in the reaction of synthesis of 7-methoxy-4-methylcoumarin, a coumarin derivative. The molecular sieves Al-SBA-1 obtained in acidic conditions proved to be ecologically friendly and selective solid state acidic catalysts for the Pechmann condensation leading to a desired coumarin derivative. Moreover, the catalysts were stable in the reaction medium so the separation of the product from the reaction environment was easier and did not require purification of the products as it did with the use of homogeneous catalysts [52].

Venkatachalam et al. [53] studied the catalytic activity in the reaction of heptanal acetalization of SBA-1 modified with aluminum or aluminum and magnesium ions. The two types of modified samples were synthesized in the same conditions, at 0 °C and in acidic environment. C_16_TEAB was used as a surfactant, TEOS as a precursor of silicon, while aluminum hydroxide and magnesium acetate were the precursors of the metals. According to the catalytic tests, the catalysts permitted obtaining similar degrees of the aldehyde conversion, irrespective of the ratios of Si/Al or Si/(Al + Mg). These observations mean that the reaction was not controlled by the catalyst acidity but by its hydrophilic-hydrophobic properties and free diffusion of the substrates and products. It is because of this free diffusion that the mesoporous materials proved to be better catalysts in the reaction of acetalization of long-chain aldehydes, than microporous materials [53].

Ji et al. [54] have succeeded in synthesizing SBA-1 modified with titanium species using tetrabutyl titanate as a metal precursor, introduced in direct synthesis. The other reagents were C_16_TEAB, TEOS and HCl, while conducting the process at 0 °C. The materials containing up to 2.5% wt. of titanium ions showed a highly ordered cubic structure and well-developed surface area higher than 1000 m^2^/g. UV-VIS (Ultraviolet-Visible Spectroscopy) analysis proved that titanium ions are well-distributed and tetrahedrally coordinated. The obtained SBA-1 materials modified with titanium ions were tested as catalysts of selective epoxidation of styrene to styrene oxide, in the presence of 30% wt. of H_2_O_2_ in acetonitrile, at 70 °C. The catalysts tested showed relatively high activity and selectivity in the above-mentioned reaction, that is, higher than the materials containing one-dimensional channels, like Ti-MCM-41 and Ti-SBA-15. A possible explanation is that the 3D cubic structure of SBA-1 is more conducive for the reagents diffusion [55].

Zhao et al. [56] have obtained a series of SBA-1 samples modified with chromium ions. They performed the synthesis at 0 °C in acidic conditions, using C_16_TEAB, TEOS and ammonium dichromate(VI) ((NH_4_)_2_Cr_2_O_7_) as the precursor of chromium, introduced by the direct method or by impregnation. The materials modified by both methods were used as catalysts for dehydrogenation of ethane with the use of CO_2_ and showed catalytic activity. Michorczyk et al. [26] have also synthesized SBA-1 modified with Cr species by the two methods, using ammonium dichromate(VI) ((NH_4_)_2_Cr_2_O_7_) as a precursor of chromium in the direct method of modification [26] and hydrated chromium(III) nitrate (Cr(NO_3_)_3_·9H_2_O) as a precursor of chromium in the impregnation method [57]. The obtained SBA-1 modified with Cr species were tested as catalysts in propane dehydrogenation with the use of CO_2_ and were found to be catalytically active in this reaction.

Srinivasu and Vinu [25] have been the first to report the synthesis of SBA-1 modified with gallium species in acidic conditions, with gallium(III) nitrate (Ga(NO_3_)_3_) as a gallium precursor. In a strongly acidic environment, at 0 °C, with C_16_TEAB as a surfactant and TEOS as a silicon precursor, they managed to obtain metallosilicates of highly ordered 3D structure of cage type and excellent textural parameters. XRD patterns and low-temperature nitrogen sorption have proved that the 3D structure of cage type 3D pores with open windows is preserved in the synthesized materials even after introduction of relatively large amounts of gallium to the silica matrix. Moreover, the specific surface area and pore volume in SBA-1 modified with gallium increased as a result of gallium ions introduction. This observation is very important from the point of view of catalysis, as these parameters determine the catalytic activity of the silicas. The use of the obtained catalysts in the reaction of *tert*-butylation of phenol revealed their very high catalytic activity, higher than that observed for example, MCM-41 modified with aluminum or iron ions [25]. The catalysts were also successfully applied in the reaction of naphthalene alkylation with propylene to obtain diisopropylnaphthalenes, being high quality solvents [58].

Imran and Maheswari [59] have synthesized SBA-1 modified with manganese, using hydrated manganese(II) acetate ((H_3_CCOO)_2_Mn·4H_2_O) as a metal precursor. They carried out the synthesis in acidic conditions at 0 °C. As follows from XRD measurements, the obtained materials showed a highly-ordered structure. The degree of ordering decreased slightly as the concentration of introduced manganese ions increased. Because of the acidity of the reaction mixture, only 30% wt. of Mn^3+^ or Mn^2+^ ions were incorporated into the silica structure, as evidenced by the ICP-MS results. The catalysts activity in oxidation of ethylbenzene with the use of *tert*-Butyl hydroperoxide (TBHP) was similar to that of Mn-MCM-41 or Mn-SBA-15 [59].

SBA-1 materials can also be modified with alkali metals species to endow them with catalytic properties. For instance Gracia et al. [60] have synthesized SBA-1 modified with Li, Na, K, Rb or Cs species, by the method of incipient wetness impregnation and applied the obtained samples as catalysts for Knoevenagel condensation between benzaldehyde or benzylacetone and ethyl cyanoacetate under conventional or microwave heating. Knoevenagel condensation is an important reaction of carbon-carbon bonds formation, used for production of fine chemicals. The main product of Knoevenagel condensation of benzylacetone and ethyl cyanoacetate is an important intermediate product in the production process of citronitrile, a compound of commercial importance because of its use in cosmetics and fragrances [61]. The SBA-1 sample modified with sodium ions was found to show a structure similar to that of unmodified silica. The sample of SBA-1 modified with sodium ions showed the highest catalytic activity in the Knoevenagel condensation, both under conventional heating and microwave heating. Moreover, in the conditions of microwave heating the yield of the reaction was higher, so it needed less energy and time [60].

#### 2.2.7. Modification of SBA-1 with Organic Functional Groups in Acidic Conditions

Besides modification with metal ions, another possibility to extend the range of application of mesoporous silicas is their modification with functional groups, introduced into the structure or on the surface of silicas. Such materials can be used for example, as adsorbents. Yoshitake et al. [62] have studied the applicability of using SBA-1 silicas modified with amino groups for the adsorption of chromates and arsenates. The amino groups from three precursors: (3-Aminopropyl)trimethoxysilane, [1-(2-aminoethyl)-3-aminopropyl]trimethoxysilane or (trimethoxysilyl)propyldiethylenetriamine were introduced onto the surface of unmodified SBA-1 by the method of grafting. The XRD patterns of the obtained materials contained three well-resolved reflections in the 2θ range 2°–3°, which evidenced highly-ordered structure of these materials. However, SBA-1 modified with amino groups had a twice smaller specific surface area than that of the unmodified sample. Functionalization with amino groups made thus modified SBA-1 silicas capable of adsorption of oxyanions (chromates and arsenates). It should be noted that the adsorption abilities of the amino-groups modified SBA-1 of 3D cubic structure was higher than that of MCM-41 materials modified in the same way [62].

Kao et al. [63] have also modified SBA-1 with amino groups from (3-Aminopropyl)trimethoxysilane (APTMS) but by the method of co-precipitation. It has been the first report on this type of synthesis. Highly-ordered and stable SBA-1 samples modified with amino groups were obtained by condensation of TMOS with APTMS in the presence of C_16_TEAB as the structure directing agent, in acidic conditions. The key factors ensuring the development of highly ordered structure of the silicas were prehydrolysis of TMOS prior to addition of the surfactant and the use of sulfuric(VI) acid instead of hydrochloric acid [63].

Kao et al. [64] have also introduced a large number of organic vinyl groups (up to 2% wt.) into SBA-1 structure in strongly acidic conditions. They obtained the material of highly ordered and stable structure, with no undesired phase transformation. The authors also studied the effect of HCl concentration, used during the extraction process, on the stability of the obtained material. They performed the synthesis at 25 °C. The vinyl groups were introduced into the material structure by the process of co-precipitation. Process of extraction was carried out on a magnetic stirrer, upon stirring an appropriate amount of the sample in a mixture of HCl and ethanol, at 50 °C for 6 h. According to the results, extraction had no destructive effect on the structure of the final materials. The presence of vinyl groups in SBA-1 made them more hydrophobic to be resistant to hydrolysis [64].

Pan et al. [65] have introduced nitrile groups into the bulk and/or on the surface of SBA-1, using C_16_TEAB, TMOS, 2-cyanoethyltriethoxysilane in strongly acidic conditions by the method of co-precipitation. Then, the nitrile groups were subjected to hydrolysis with the use of acids (HCl or H_2_SO_4_) as catalysts of this process in order to investigate the effect of the acid type on the –CN group behavior in SBA-1 material. It has been shown that transformation of –CN groups into –COOH under the effect of acid did not affect the synthesis of SBA-1. Moreover, the majority of cyanide groups remained in the unchanged form when the reaction took place in a short time in the presence of HCl in a low concentration. Almost all –CN groups were transformed in –COOH under the effect of H_2_SO_4_ when the reaction time was longer [65].

Tsai et al. [66] have obtained highly-ordered and stable SBA-1 modified with sulfonic groups, by the method of co-precipitation. The precursor of –SO_3_H was MPTMS ((3-Mercaptopropyl)trimethoxysilane) containing –SH groups oxidized by H_2_O_2_ to the desired functional groups. The whole process of synthesis took place in one-stage. To the reaction mixture of C_16_TEAB and HCl, a mixture of TEOS and MPTMS was added, then the mixture was stirred at 0 °C. Subsequently, the mixture was heated to room temperature and H_2_O_2_ was added on stirring. The precipitate was subjected to hydrothermal treatment at 100 °C, filtered off without washing and dried. Surfactant was removed from the pores of the material by extraction with a mixture of HCl and ethanol. It has been proved by XRD results that introduction of different amounts of H_2_O_2_ (maximum molar ratio H_2_O_2_/MPTMS was 11) into the reaction mixture did not disturb the structure of SBA-1. The diffractograms of the obtained modified silicas showed three well-resolved reflections, indicating a highly-ordered cubic structure of Pm$\bar{3}$n symmetry, so the applied synthesis of SBA-1 modified with sulfonic groups was proved effective [66].

#### 2.2.8. Application of Unmodified SBA-1 Obtained in Acidic Conditions

**Figure 5:**
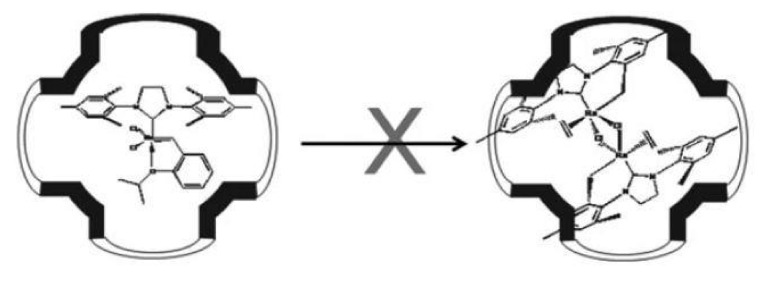
Proposed mechanism preventing dimerization of ruthenium complex in nanocages of SBA-1. Reprinted from Reference [67] with permission from The Royal Society of Chemistry.

Mesoporous SBA-1 material was used as a solid olefin metathesis catalyst after the introduction of Hoveyd-Grubbs catalyst, which is a ruthenium complex, into its regular three-dimensional structure [67]. The obtained catalyst showed much higher recycling ability than other mesoporous materials in which the Hoveyd-Grubbs catalyst, for example, SBA-15, SBA-16 or MCM-41, was placed. The extraordinary recycling capacity can be attributed to isolated nanocages present in the SBA-1 material of appropriate size, which can effectively prevent dimerization of the ruthenium complex due to space limitations (Figure 5) [67]. Li et al. [68] have used SBA-1 for obtaining highly-ordered carbon materials of Pm$\bar{3}$n symmetry.

#### 2.2.9. Synthesis of SBA-1 in Basic Conditions

As follows from the above-presented literature data, the SBA-1 materials obtained in strongly acidic conditions showed low stability upon washing with water. Attempts have been made at improving the stability of these materials by increasing the amount and concentration of the added acid or by addition of auxiliary compounds (D-fructose, alcohols). A range of applications of SBA-1 silicas requires their modification with metal ions or functional groups. Unfortunately, in strongly acidic conditions incorporation of metals to SBA-1 proved little effective as evidenced by low metal loading and formation of additional metal structures instead of isomorphous replacement of silicon atoms [56,69].

In order to improve the silicas stability and effectiveness of their modifications, attempts have been made to synthesize them in basic conditions. Liu et al. [69] have been the first to synthesize SBA-1 in basic conditions, at pH close to 9. They used sodium silicate as a precursor of silica, C_16_TEAB as a surfactant and performed the reaction in the presence of NaCl. The silicas obtained revealed a number of advantages over those synthesized in strongly acidic conditions. First of all, they were resistant to washing with water and to hydrothermal processing. Their high stability stemmed from the fact that in basic conditions silicon condensation is more complete. Moreover, the synthesis in basic conditions is more cost-effective and environmentally friendly than that performed with large amounts of inorganic acids and TEOS as a precursor of silicon. Introduction of aluminum ions into SBA-1 in basic conditions was more effective than in acidic conditions and the catalyst obtained was active for alkylation of 2, 4-Di-*tert*-butylphenol with cinnamyl alcohol [69].

Lin et al. [70] have undertaken the synthesis of SBA-1 modified with aluminum ions in basic conditions, using a zeolite modified with aluminum as a precursor of silicon and aluminum (zeolite Al-ZSM-5). The obtained SBA-1 silicas modified with that zeolite and aluminum ions showed highly-ordered pore structure as proved by the three reflections in the XRD diffractograms in the range of small 2θ, corresponding to the planes (200), (210) and (211), characteristic of Pm$\bar{3}$n symmetry. No separate zeolite phase was formed as no reflections corresponding to ZSM-5 zeolite structure, that would have been in the range of wide 2θ, were observed. The obtained silicas proved to be effective catalysts in Friedel-Crafts alkylation of 2, 4-Di-*tert*-butylphenol with cinnamyl alcohol. The catalysts obtained were noted to combine the benefits of zeolites (strong acidity) and mesoporous materials (diffusion of reagents) [70].

**Figure 6:**
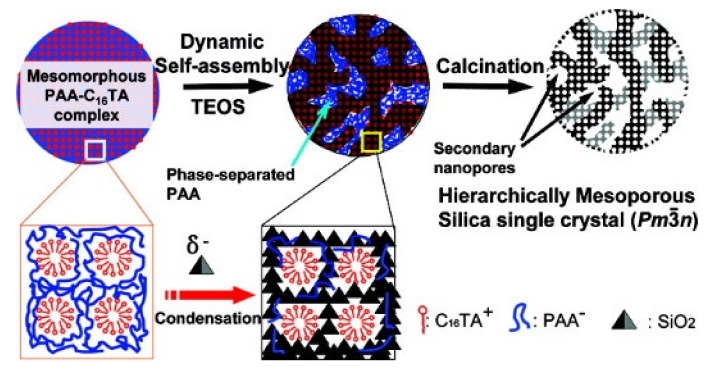
Schema of the synthesis of hierarchic SBA-1 silica with the use of PAA/C_16_TAB complex. Reprinted with permission from Reference [71]. Copyright (2010) American Chemical Society.

Wang et al. [71] have also synthesized SBA-1 in basic conditions using poly(acrylic acid) (PAA), C_16_TAB and NH_4_OH for setting the pH of the reaction mixture to be 10–11. At the desired pH the mixture assumed the color of milk indicating the formation of polyelectrolyte-surfactant (PAA/C_16_TAB) complex. Then TEOS was added. The hydrolysis process of silica precursor was carried out at room temperature, whereas the temperature of ageing of the reaction mixture (from 25 °C to 120 °C) was modified. The results obtained permitted proposing a mechanism of formation of the hierarchic nanoporous SBA-1 silicas. Thanks to the use of the complex PAA/C_16_TAB as a template, a monocrystalline and mesoporous SBA-1, containing secondary nanopores, was obtained (Figure 6). Importantly, the presence of a large number of secondary nanopores did not disturb the monocrystalline mesostructure.

In aqueous solution, as a result of co-organization of PAA and C_16_TAB micelles, a mesomorphic PAA/C_16_TAB complex was formed. Hydrolysis of added silica precursor (TEOS) caused formation of negatively charged silica oligomers (at pH = 10–11), which co-organized with mesomorphic PAA/C_16_TAB complexes. The electrostatic interactions between PAA and C_16_TAB were disturbed by negatively charged silica oligomers, while PAA chains were separated from the complexes forming phase separated PAA domains, which can serve as templates for secondary (interstitial) nanopores inside SBA-1 after the calcination process [71].

On the basis of the mechanism proposed, Li et al. [72] have synthesized, in a basic environment, stable SBA-1 silicas modified with aluminum ions, which showed a hierarchic structure (with secondary mesopores). As a template they used a complex formed of poly(acrylic acid) and a cationic surfactant—hexadecylpyridinium chloride (CPC). The materials were characterized by a large surface area, large pore volume and high hydrothermal stability. The use of this catalyst in Friedel-Crafts alkylation of toluene and benzyl alcohol revealed its high activity, thanks to the acidic centers built in the structure (aluminum ions) and hierarchical mesoporous structure.

Lin et al. [73] have been the first to obtain, in basic conditions, SBA-1 silicas modified with titanium. Basic conditions helped incorporation of Ti^4+^ ions into the silica structure. Moreover, according to the elemental analysis and ICP-MS results, the amount of titanium ions introduced into the silica was closed to that assumed to be present in the reaction mixture. The modified silicas showed high catalytic activity in oxidation of 2, 3, 6-trimethylphenol, higher than that of the catalysts with 1D channels (Ti-MCM-41, Ti-SBA-15).

#### 2.2.10. Applications of SBA-1 Silicas Synthesized in Basic Conditions

**Figure 7:**
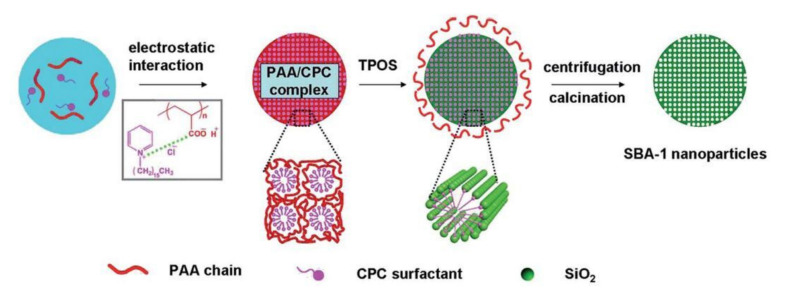
Schematic presentation of the procedure for obtaining SBA-1 nanoparticles using PAA/CPC complex as a template. Reprinted from Reference [75] with permission from The Royal Society of Chemistry.

In the materials with 1D porous structure, the diffusion of large molecules can be hindered by blocking the pores at the entrances. However, these limitations do not hold for the silicas with 3D porous structures. Thanks to its 3D structure of cage pores joined through open windows, SBA-1 is a promising support for enzyme immobilization [74]. The possibility of enzymes immobilization is to a high degree dependent on the pore size of the mesoporous support. Attempts have been made at the synthesis of SBA-1 silicas of larger pores. Xu et al. [75] have proposed a simple synthesis of monodisperse nanoparticles of SBA-1 of cubic structure and Pm$\bar{3}$n symmetry. They used poly(acrylic acid) (PAA) complex with hexadecylpyridinium chloride (CPC) as a template and tetrapropyl orthosilicate (TPOS) as a precursor of silicon, in basic conditions. The pore size was enlarged by using 1, 3, 5-trimethylbenzene (TMB). Depending on the amount of TMB introduced, the pore diameter in SBA-1 increased from 2.5 nm (for the material synthesized without TMB) to 5.3 nm, while the particles morphology showed insignificant changes. The obtained nanoparticles of silica of highly-ordered pore structure and enlarged pore size were used for immobilization of lysozyme. According to the results, the enzyme immobilized inside the pores retained its activity and was resistant to leaching. In view of the above, SBA-1 silicas are promising supports for biosensors, for application in biocatalysis and in drug delivery systems [75]. The scheme of obtaining SBA-1 nanoparticles is shown in Figure 7.

PAA and CPC molecules connect with each other through electrostatic interactions, resulting in a mesomorphic PAA/CPC complex. In the next stage of the synthesis, TPOS is added. During TPOS hydrolysis (slower rate than TEOS hydrolysis), negatively charged silica oligomers are formed, which slowly penetrate into mesomorphic PAA/CPC complexes as a result of strong binding of CPC surfactant with silica oligomers. On the other hand, the electrostatic repulsion force between the silica oligomers and the polyelectrolyte chains caused the breakaway of PAA/CPC complexes from its chains, which penetrated into the aqueous phase. SBA-1 nanoparticles were obtained after filtration/centrifugation of the formed colloidal dispersion followed by calcination process [75].

Due to the high potential of SBA-1 materials for enzyme immobilization, Saikia et al. [76] have applied SBA-1 modified with carboxyl groups for immobilization of papain. In the synthesis TEOS and carboxyethyl silanetriol sodium salt (CES) as silicon precursors, poly(acrylic acid) (PAA) complex with hexadecylpyridinium chloride (CPC) as a template and trimethylbenzene as the pore-enlarging agent were used. Thanks to the use of CES, SBA-1 silica was additionally modified with –COOH groups. As they were the active centers in the mesopores, the modified silica can be used for a wider range of applications. At pH of 8.2, the obtained modified SBA-1 showed extraordinarily high adsorption of papain. Of importance in this phenomenon are the pore size and electrostatic attraction between carboxylic groups and papain molecules. Immobilized papain shows better properties than free one, that is the former is characterized by higher thermal stability, tolerance of a greater range of pH and higher thermal stability [76].

Environmental pollution by toxic dyes causes a serious threat to humans and living organisms. Mesoporous silica materials, characterized by a well-developed porous structure and specific surface area, as well as the possibility to modify their surface using organic functional groups, show great potential for use as adsorbents for dyes. Therefore, Lin et al. [77] have synthesized bifunctional mesoporous SBA-1 silicas, modified with carboxylic and amino groups, in order to apply them for adsorption of toxic anionic or cationic dyes. They used PAA/CPC complex as a template and TEOS as a silicon precursor and performed the synthesis in basic conditions. The carboxyl groups were introduced by the co-precipitation with the use of CES, while the amino groups were incorporated with the use of APTMS by grafting onto the pure or carboxyl groups modified SBA-1 silica. The SBA-1 modified only with carboxyl groups were found to show strong affinity to cationic dyes at high pH because of the beneficial effect of electrostatic interaction between the cationic dye and –COO^−^ groups. The SBA-1 silicas modified with amino groups exhibited strong ability to adsorb anionic dyes at low pH, because of the interactions of –NH_3_^+^ groups with anionic dyes. Particularly promising for selective removal of a given dye from a mixture of cationic and anionic dyes proved to be SBA-1 modified with both carboxyl and amino groups [77].

## 3. SBA-2 Silicas

### 3.1. General Characterization of SBA-2 Silicas

SBA-2 type silicas have not been so thoroughly described as SBA-1. The former have a 3D pore network made of spherical cavities ordered in hexagonal close-packed (hcp) system and cubic close-packed system (ccp), joined through cylindrical channels [78,79,80]. It should be emphasized that the structure of SBA-2 silicas is similar to that of SBA-12 showing 3D hexagonal structure and P6_3_/mmc symmetry. The latter have been obtained using triblock copolymer as a mesoporous structure directing compound [81,82]. After the first synthesis of SBA-2 silica, only the hexagonal pore system was identified in its structure, while the presence of the cubic system of pores was found later as a result of detailed investigation [83]. The hitherto literature has provided only a few applications of these silica materials in catalysis and adsorption, as described below.

### 3.2. Syntheses and Structural Characterization of SBA-2 Silicas

SBA-2 type materials were for the first time synthesized by Huo et al. in 1995, so a year later than SBA-1 silicas [29]. The materials were synthesized with the use of gemini surfactants C_n_H_2n+1_N^+^(CH_3_)_2_(CH_2_)_s_N^+^(CH_3_)_2_C_m_H_2m+1_, labelled as C_n-s-m_ and silica precursor_,_ in either acidic or basic environment. Thanks to the use of dicationic templates, the mesoporous structure can be modified by changing the length and character of the side chain as well as the type of the linking group. The terminal fragment of the surfactant, C_n-s-1_, plays a particular role in obtaining the 3D hexagonal cage structure of SBA-2. On the basis of XRD and electron diffraction data, Huo et al. have proposed a model of SBA-2 type structure formation. In this model, spherical micelles were in hexagonal close-packing and between them amorphous silica walls were formed. Removal of the template by calcination at 500–600 °C resulted in obtaining mesoporous cage structure [29,48]. XRD patterns of these materials after calcination showed reflections in the 2θ range from 2° to 7°, corresponding to the following planes (100), (002), (101), (110), (103), (112), (211). The specific surface area and pore size were measured by the low-temperature nitrogen sorption to be 609 m^2^/g and 3.5 nm, respectively [29].

**Figure 8:**
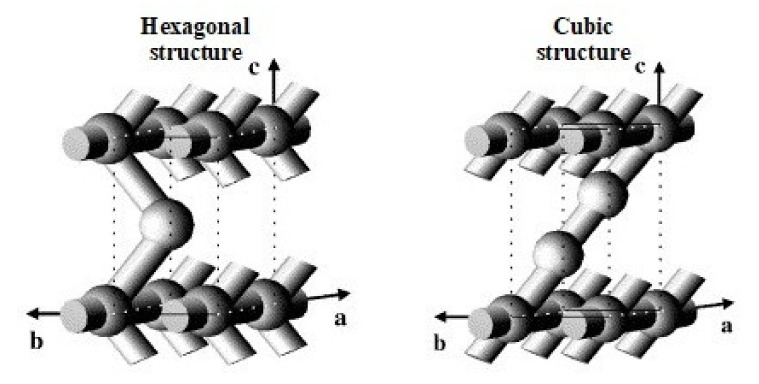
The network of pores in SBA-2. Reprinted with permission from Reference [84] with minor modification. Copyright (2003) Elsevier B.V.

Further studies of the structure of SBA-2 with the use of high-resolution transmission electron microscopy (HRTEM) have been undertaken by Zhou et al. [83]. The results confirmed the model of close packing of micelles and revealed some details on spatial connection of the pores, suggesting its 2D nature. According to these authors the pore system in SBA-2 is composed of two types of subsystems. Type 1 is made of straight mesopores arranged along the *axis a*, while type 2 is made of zigzag mesopores arranged in the direction [001] linking selectively the supercages along the *axis c* [83]. Thus, the SBA-2 structure can be considered as the intergrowth of two different structures: hexagonal and cubic (regular), which contain a complex set of narrow channels connecting large cavities—Figure 8 [84].

Hunter and Wright [85], have studied the effects of pH, temperature and type of gemini surfactant on the product of synthesis of SBA-2. They have proved that SBA-2 structure is formed in temperatures from RT to 100 °C. The unit cell parameter depends on the size of the molecule of gemini surfactant used and on pH of the reaction mixture, that can be varied from 10 to 12. Introduction of aluminum ions by direct synthesis into SBA-2 structure, at the Si/Al ratio of 11, resulted in getting materials of the textural parameters similar to those of unmodified SBA-2 but of poorer structural ordering [85].

Hunter et al. [86] have studied the morphology and microstructure of SBA-2. This material synthesized with the use of CH_3_(CH_2_)_15_N(CH_3_)_2_(CH_2_)_3_N(CH_3_)_3_Br_2_(C_16-3-1_), at basic pH and at room temperature has particles of three morphologies: small solid balls, flat plates and larger empty spheres. The small solid balls appeared as a suspension, the empty spheres formed when the silica structure built up around the air bubbles, while the plates formed at the solvent/air interface or as a result of breaking up of the empty spheres. At high speed of the reaction mixture stirring, the number of empty spheres decreased to the advantage of appearance of a greater number of plates. The HRTEM images revealed a high degree of disordering in SBA-2, which explains the lack of crystalline morphologies of the particles observed in other 3D ordered materials. The disordering is a result of irregular arrangement of domains within the balls and plates as well as a within the domains. The XRD patterns in the range of small 2θ have shown no distinct maxima characteristic of the hexagonal phase for some samples, so in these samples the cubic phase was dominant [86]. Garcia-Bennett et al. [82] have synthesized SBA-2 at low temperature of −4 °C, either in acidic or basic environment, with the use of C_16-3-1_ surfactant. They proved that in basic conditions SBA-2 phase, made of close packed system of micelles, was obtained, while in acidic conditions a mesoporous SBA-1 silica accompanied by a family of SBA-2 silicas, made of close packed balls, were obtained.

**Figure 9:**
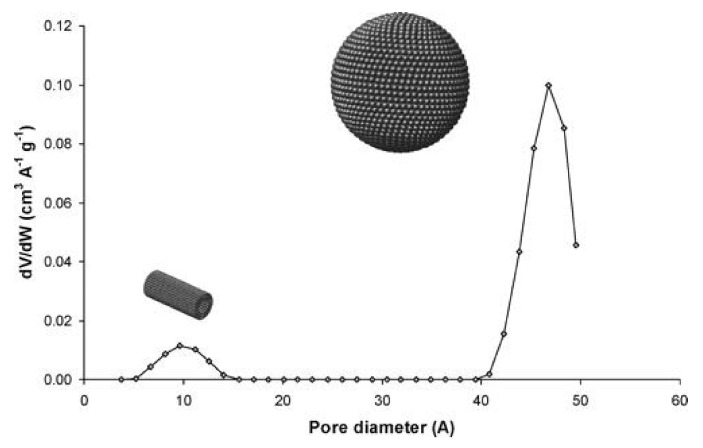
Pore size distribution in SBA-2 obtained by the results of Pèrez-Mendoza. Reprinted with permission from Reference [87]. Copyright (2004) American Chemical Society.

Pèrez-Mendoza et al. [87] studied the types of connections in the porous structure of SBA-2 by adsorption of methane and ethane under elevated pressure in these materials. The obtained data were confronted with the results of Monte Carlo simulations for the models of different complexity. On the basis of the fit of the simulation data to the experimental results it was concluded that the model assuming the presence of spherical cavities without interconnecting channels was incorrect. To get an acceptable fit it was necessary to take into account the size of the cavities and channels. Thanks to the pore size distributions obtained on the basis of the fit, some new information was obtained about the structure of SBA-2. The channels joining the cavities were estimated to have diameters in the range 0.5–1.5 nm, while the cavity diameter was 4–5 nm [87]. The pore size distribution obtained by these authors is presented in Figure 9.

More detail information on the geometry and connections in the pore network has been provided by further studies of Pèrez-Mendoza et al. [78], who carried out a percolation analysis of the adsorption of ethane and nitrogen in the pore system of SBA-2. The experimental data interpreted together with the results of Monte Carlo simulation provided new information on the structure of SBA-2. The effective coordination number of cavities defined as the average number of channels per cavity, large enough to permit the flow of nitrogen, was 4.9, whereas taking into accounts channels large enough to allow ethane to pass—1.8 [78].

To understand the effect of the calcination process on pore size change, Pèrez-Mendoza et al. [88] studied the adsorption of nitrogen, *n*-butane and isobutane with the use of mesoporous silica SBA-2. The synthesized SBA-2 silicas were calcined at 700 °C, 800 °C and 900 °C. Analysis of XRD patterns and HRTEM images has shown that all obtained materials had structures characteristic of SBA-2 of P6_3_/mmc symmetry. With increasing calcination temperature, the size of the elementary cell decreased. The reduction of the unit cell size was also visible in the nitrogen sorption isotherms. With increasing temperature of calcination the number of cavities available for nitrogen molecules decreased, which implies that in the samples subjected to calcination at above 700 °C, some channels were narrowed or even closed. However, the pore size distribution indicated that the sample calcined at 700 °C showed smaller cavities and lower total pore volume than the sample subjected to extraction with the solvent. For the samples calcined at 800 °C and 900 °C, the total pore volume decreased but the average size of the cavities remained the same. Thus, the reduced volume available to nitrogen in the samples calcined at higher temperatures was related to the availabilities of the cavities to nitrogen and not to the reduced size of the cavities. According to the results of adsorption/percolation of hydrocarbons, the network of pores in the sample calcined at 700 °C was available to both isomers, while in the sample calcined at 800 °C adsorption of isobutane is insignificant but that of *n*-butane is high. For the sample calcined at 900 °C, adsorption of both hydrocarbons was slight. The above-presented results suggest that by the choice of calcination temperature it is possible to tune the availability of different adsorbents to the mesoporous structure of SBA-2 in order to achieve a molecular-sieving effect [88].

Ferreiro-Rangel et al. [89] have performed a kinetic Monte Carlo simulation in order to mimic the stages of condensation, aggregation, deformation and calcination during the synthesis of SBA-2. Their results suggest that the pores are joined through the windows formed in the process of micelles aggregation, induced by close packing of spherical micelles and the presence of water molecules at the silica/micelle interface. The pore models obtained as a result of the simulations are comparable with the experimental data on the pore systems and nitrogen sorption. This consistence means that the applied method of Monte Carlo simulation can be applied for modelling of mesoporous silicas, even those of complex structures [89]. On the basis of the simulation of adsorption on SBA-2 pore system Ferreiro-Rangel et al. [90] proved that the roughness of the SBA-2 materials is of key importance for prediction of adsorption behavior at low pressure, which is of importance for practical use.

Analysis of the pore size distribution and their availability in SBA-2 as well as in SBA-1 silicas was the subject of interest for Zapilko and Anwander [91]. They have silylated the surfaces of the silicas studied with two silylamines of different particle sizes—tetramethyldisilazane (HN(SiHMe_2_)_2_) and tetramethyldiphenyldisilazane (HN(SiMe_2_Ph)_2_). According to their results the pores of SBA-1 silicas are accessible to both smaller particles of for example, HN(SiHMe_2_)_2_ and larger of for example, HN(SiMe_2_Ph)_2_. However, the pores of SBA-2 silicas were found accessible to HN(SiHMe_2_)_2_ but too small for HN(SiMe_2_Ph)_2._ That is why the reaction with HN(SiMe_2_Ph)_2_ was limited to the silica surface as the silanol groups present inside the pores were not functionalized. In conclusion, such reactions are very helpful for design of new catalysts of a cage type structure whose selectivity would be based on the size of molecules taking part in the reaction [91].

### 3.3. Application of SBA-2 Materials

Literature provides just a few papers on modification of SBA-2 silicas with organic functional groups or metal ions and the use of such materials. Díaz et al. [92] have synthesized SBA-2 silicas modified with thiol groups from 3-mercaptopropyltriethoxysilane (MPTES). The synthesis of SBA-2 modified with thiol groups was performed by the method of coprecipitation, in basic environment and at room temperature, with the use of a gemini surfactant C_16-3-1_, that after the synthesis was removed by extraction with a mixture of ethanol and HCl. XRD results proved that after the extraction the modified silicas had poorly ordered structure because of the disturbing effect of the introduced functional groups. A large number of defects disturbing the ordering of the channels and cavities resulted in almost total blocking of pores in the process of extraction. The specific surface area of silica modified with thiol groups was below 10 m^2^/g. The oxidation of –SH to –SO_3_H with H_2_O_2_ was ineffective, which can be related to the inaccessibility of channels. The use of the synthesized silicas modified with thiol groups subjected to oxidation in the reaction of esterification of glycerol with oleic acid at 120 °C has brought low conversions of the fatty acid and low selectivity to the desired products, so the modified SBA-2 silicas showed low catalytic activity [92]. Similar studies performed by Pérez-Parient et al. [84] who tested SBA-2 modified with sulfonic groups in esterification of glycerol with fatty acids (lauric or oleic) proved very low catalytic activity of the material studied. The sulfonic groups introduced into the SBA-2 structure or on its surface blocked the pores [84].

Mesoporous materials of hierarchic porous structure are much promising because of the availability of active centers and increasing the rate of diffusion. Similarly as for SBA-1 materials, the hierarchic porous structure can be induced through the use of a complex of a polyelectrolyte and surfactant [71]. Shi et al. [79] have been the first to synthesize SBA-2 with hierarchic porous structure modified with titanium ions, using as a template an organic mesomorphic complex of anionic poly(acrylic acid) and hexadecyltrimethylammonium bromide (C_16_TAB). The titanium precursor was TiOSO_4._ The obtained material showed a highly-ordered 3D cubic structure of Pm$\bar{3}$n symmetry or a 3D hexagonal structure of P6_3_/mmc symmetry, the presence of larger secondary mesopores and a high content of titanium, up to 5% wt. At that time it has been the highest content of titanium introduced by direct synthesis to silica materials obtained. SBA-2 modified with titanium ions showed excellent catalytic activity in oxidizing desulfurization of Diesel oil at low temperatures (24 °C or 40 °C) [79].

Thanks to very good sorption properties and chemical stability, mesoporous silicas are very good selective adsorbents of volatile organic compounds (VOC) [93]. Emparan-Legaspi et al. [94] have synthesized SBA-2 and used it as adsorbent for separation of a mixture of benzene/cyclohexene by dynamic adsorption. SBA-2 was synthesized with the use of TMAOH, gemini surfactant (CH_3_(CH_2_)_15_N(CH_3_)_2_(CH_2_)_3_N(CH_3_)_3_Br_2_), TEOS, at room temperature. The surfactant was removed by extraction with a mixture of HCl/ethanol. At the next stage the obtained SBA-2 samples were subjected to calcination at 240 °C, 550 °C or 800 °C. The material calcined at 240 °C showed the greatest adsorption capacity, while that calcined at 550 °C showed the highest selectivity coefficient. The material calcined at 800 °C did not show adsorption properties, so it can be concluded that the thermal treatment at high temperatures results in the pore narrowing which then become inaccessible to the hydrocarbons studied [94].

## 4. HMM Type Materials

### 4.1. General Characterization of HMM Type Materials

In 1999 Inagaki et al. for the first time synthesized a new group of mesoporous organic-inorganic hybrid materials or hybrid mesoporous materials, labelled by the acronym HMM [95]. The materials belong to Periodic Mesoporous Organosilicas (PMOs) as they contain in their structure organic and inorganic groups making a hybrid organic-inorganic lattice linked by covalent bonds [95]. The HMM group is divided into HMM-1 and HMM-2 subgroups. Their structure is built of uniform ethyl fragments (–CH_2_–CH_2–_) and silica groups (Si_2_O_3_), making a network joined through covalent bonds [96,97,98]. The porous structure of these materials is completely different than that in mesoporous materials built of an inorganic lattice with organic modifiers grafted on the surface. HMM-1 and HMM-2 show highly-ordered mesoporous structure and well-defined morphology of hexagonal rods and spherical particles, respectively [96]. These two materials were synthesized using the same reagents: 1, 2-Bis(trimethoxysilyl)ethane (BTME) and octadecyltrimethylammonium chloride (ODTMACl), in basic conditions. Their structure was controlled by the temperature of synthesis and molar ratio of the components of the synthetic mixture. The removal of surfactant by extraction with a solvent opened the uniform pores in the materials and it did not deteriorate the ordered structure. The materials were characterized by hydrothermal stability [95,99]. HMM-1 showed a 2D hexagonal structure of p6mm symmetry with 1D pores of diameters smaller than 10 nm and well-developed surface area reaching even 1000 m^2^/g. Thanks to these properties HMM-1 was applied as a template for the synthesis of nanoparticles and nanowires of metals [100,101]. HMM-2 of a 3D structure of P6_3_/mmc symmetry found similar applications [102].

### 4.2. Synthesis of HMM Materials

**Figure 10:**
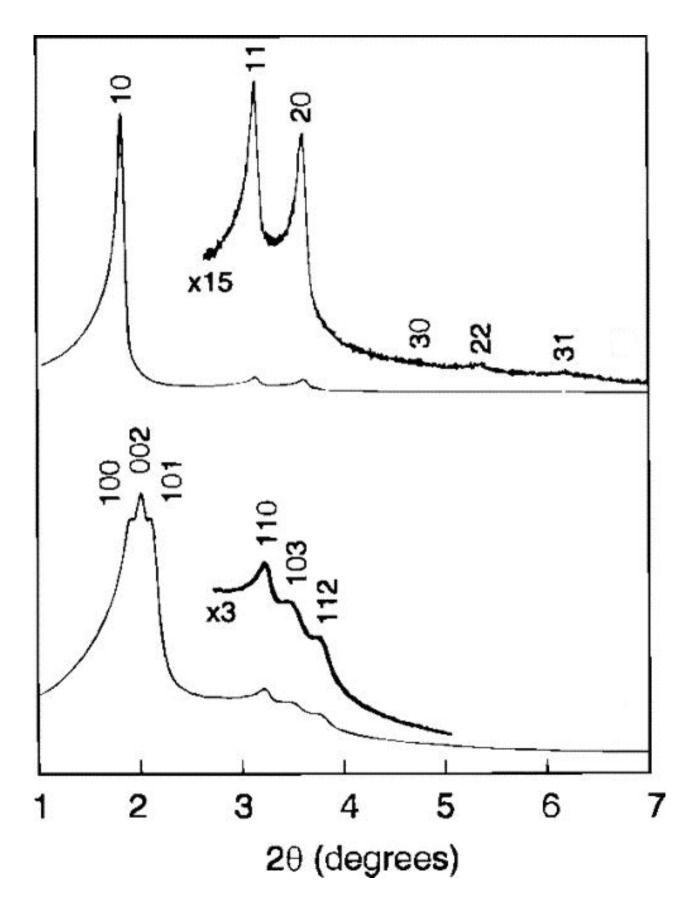
Diffractograms of HMM-1 (top) and HMM-2 (bottom). Reprinted with permission from Reference [95]. Copyright (1999) American Chemical Society.

HMM materials are synthesized using 1, 2-Bis(trimethoxysilyl)ethane (BTME) as a source of silicon. In this precursor the ethyl fragment (–CH_2_–CH_2_–) is localized between two trimethoxysilyl groups (Si(OCH_3_)_3_). This compound has been earlier used for the synthesis of amorphous porous hybrid materials [95]. Prior to the HMM synthesis, BTME was obtained in the reaction of 1, 2-Bis-(trichlorosilyl)ethane with sodium methanolate (NaOCH_3_) and anhydrous methanol. To a mixture of octadecyltrimethylammonium chloride (ODTMACl, C_18_H_37_N(CH_3_)_3_Cl), sodium hydroxide (NaOH) and water, BTME was introduced and the obtained mixture was stirred at 25 °C. The molar ratio of reagents determined the final structure of HMM. When the molar ratio of the reagents was 1 BTME:0.12 ODTMACl:1 NaOH:231 H_2_O, a white precipitate appeared immediately after addition of BTME, then the stirring was continued at 25 °C for 24 h. When the molar ratio was 1 BTME:0.57 ODTMACl: 2.36 NaOH:353 H_2_O, the white precipitate did not appear immediately but when temperature was increased to 95 °C, after stirring for 14 h at 25 °C. That is why the suspension was maintained at 95 °C for 21 h. The suspension were filtered off, washed with water and dried. Surfactant was removed from the pores by extraction with a mixture of ethanol or water with HCl, at 50 °C for 6 h. The synthesized materials were subjected to structural investigation by a few methods. The results revealed that two types of HMM materials were obtained, whose diffractograms are presented in Figure 10. One of them, HMM-1, showed a 2D hexagonal structure as evidenced by XRD reflections characteristic of this type of structure, corresponding to the planes (100), (110) and (200). Apart from these reflections there were also ones indicating a high-ordering of mesoscopic pores. SEM images revealed the presence of rod-like particles of hexagonal cross-section, while HRTEM images indicated the presence of a hexagonal system of uniform pores. HMM-2 showed a 3D hexagonal structure evidenced by the XRD reflections corresponding to the planes (100), (002), (101) as well as (110), (103) and (112) for the Bragg angle ranging from 2° to 4°. The structure of HMM-2 proved to be of higher ordering than those of SBA-2 or SBA-12 of 3D hexagonal structure. SEM images revealed spherical shapes of the HMM-2 particles. The low-temperature nitrogen sorption measurements permitted determination of the pore size and specific surface area of HMM-1 and HMM-2 silicas to be 3.1 nm, 750 m^2^/g and 2.7 nm, 1170 m^2^/g, respectively. The above-presented results have shown that the synthesis based on the polymerization of organosilane, whose monomer contained two alkoxyl groups, in the presence of an appropriate surfactant led to formation of two types of mesoporous materials of highly-ordered structure [95].

Kruk and Jaroniec [99] have studied the materials obtained by the above-described method, HMM-1 and HMM-2, by nitrogen and argon adsorption and by thermogravimetric method (TGA). The results implied that mesoporous structure of HMM-1 was higher ordered than that in MCM-41 of hexagonal structure and p6mm symmetry, while HMM-2 showed a higher-ordering of mesoporous structure, greater adsorption abilities and larger specific surface area than other mesoporous ordered materials of 3D structure. Moreover, HMM-1 and HMM-2 materials were proved to have not much different surface properties, although the interactions of the HMM-2 surface with adsorbates were weaker, which may be related to the localization of the ethyl fragments in the silica structure. TGA results confirmed that surfactant had been completely removed from the pores of these materials [99].

### 4.3. HMM Modifications and Applications

Dhepe et al. [103] have synthesized HMM-1 materials modified with sulfonic groups by direct synthesis or by grafting. The modified silicas were applied as solid catalysts for the reaction of hydrolysis of saccharose or starch. The materials obtained with the use of BTME or BTMB (1, 4-Bis(triethoxysilyl)benzene) and MPTMS as a source of –SH groups that were later oxidized to –SO_3_H groups, showed highly-ordered hexagonal structure, large specific surface area and high catalytic activities in the reactions of hydrolysis. Their catalytic activities were much higher than those of the commercial catalysts Amberlyst-15 and Nafion SAC-13. The reuse of the catalyst obtained with BTMB and modified with MPTMS by direct synthesis did not reduce its activity after three reaction cycles. Moreover, no leaching of the functional groups was noted. On top of that the highly-ordered hexagonal structure of the catalyst was maintained after the reactions [103].

HMM materials modified with metal ions have been used as catalysts for hydrogenation of light alkanes. Dhepe et al. [98] have synthesized bimetallic and monometallic catalysts supported on HMM-1 modified with rhodium and platinum or only rhodium, using supercritical carbon(IV) oxide (scCO_2_) during the synthesis. HMM-1 was impregnated with a solution containing the metal precursors, that is, rhodium(II) acetate dimer ([Rh(OAc)_2_]_2_) and platinum(II) acetylacetonate (Pt(acac)_2_) dissolved in tetrahydrofuran (THF). The mixture was stirred for 16 h, then the solvent was evaporated and dried under vacuum. Half of the obtained product was subjected to supercritical carbon(IV) oxide (scCO_2_), both samples were subjected to calcination and reduction in a stream of hydrogen. The sample containing only rhodium was prepared according to the same procedure, of course without adding the platinum precursor. The sample subjected to scCO_2_ were found to have metal particles of smaller diameter and better dispersed than in the sample not subjected to scCO_2_. Moreover, the metal particles in the samples treated with scCO_2_ were placed inside the channels of HMM-1, while in the samples not treated with scCO_2_, the metal particles were on the outer surface of the silica support. The catalysts obtained were tested in the reaction of hydrogenation of *n*-butane. The materials treated with scCO_2_ showed higher catalytic activity and higher selectivity to ethane than those not treated with scCO_2_. Moreover, the performance of bimetallic catalysts was superior to that of those containing only rhodium species, as the addition of platinum ions helped preventing multiple hydrogenation caused by hydrogen activation [98].

**Figure 11:**
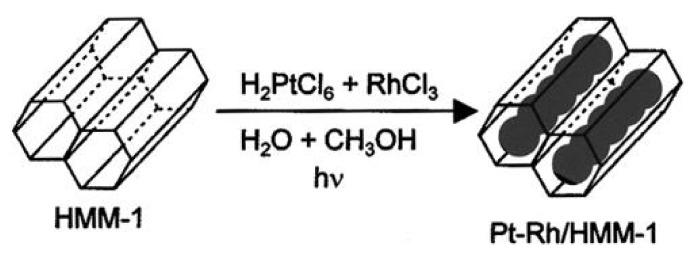
Typical synthesis of metal nanowires inside the channels of HMM-1. Reprinted with permission from Reference [104]. Copyright (2001) American Chemical Society.

HMM materials were applied as matrices for the syntheses of nanowires and metal nanoparticles. Fukuoka et al. [104] have reported the synthesis of new bimetallic nanowires obtained from platinum and rhodium (Pt-Rh), platinum and palladium (Pt-Pd) as well as monometallic nanowires of platinum or rhodium. The first stage was the synthesis of HMM-1, according to the procedure described by Inagaki et al. [95]. Then the material was impregnated with chloroplatinic acid hexahydrate (H_2_PtCl_6_·6H_2_O) or rhodium(III) chloride hydrate (RhCl_3_·3H_2_O). The dried material was subjected to water and methanol, then irradiated with radiation from a high-pressure mercury lamp at room temperature for 24 h, then dried (Pt-Rh/HMM-1). A schema of the procedure is given in Figure 11. The same method was applied to obtain a sample containing platinum and palladium, their sources were H_2_PtCl_6_·6H_2_O and H_2_PdCl_4_. Analysis of TEM images revealed that the HMM-1 channels of 3.1 nm in diameter contained Pt-Rh nanowires of 3 nm in diameter and average length of 23 nm, Pt-Pd nanowires of average length of 170 nm and monometallic Pt or Rh nanowires of average lengths of 120 nm and 48 nm, respectively. HRTEM images showed that bimetallic as well as monometallic nanowires were composed of joined metal nanoparticles in a necklace-shaped morphology. It should be noted that according to XRD diffractograms in the small angle range, the materials had highly-ordered structure that did not change significantly as a result of photoreduction. Interestingly, the Pt-Pd nanowires were found to show unique magnetic properties [104].

**Figure 12:**
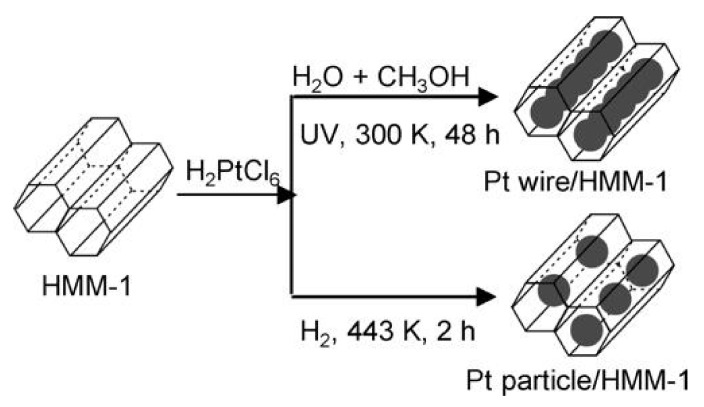
Schematic presentation of the synthesis of Pt nanowires and nanoparticles in HMM-1. Reprinted with permission from Reference [106]. Copyright (2004) American Chemical Society.

Fukuoka et al. [105,106] have studied the mechanism of platinum nanowires formation inside the HMM-1 pores. This material was impregnated with H_2_PtCl_6_·6H_2_O and then subjected to reduction by two methods. According to the first method, steam and methanol vapor were adsorbed on the impregnated material and then reduction was performed using a high-pressure mercury lamp. According to the second one, the material impregnated with a metal solution was subjected to reduction with gas hydrogen. The first method led to Pt nanowires, while the second brought Pt nanoparticles. The procedure is schematically shown in Figure 12.

**Figure 13:**
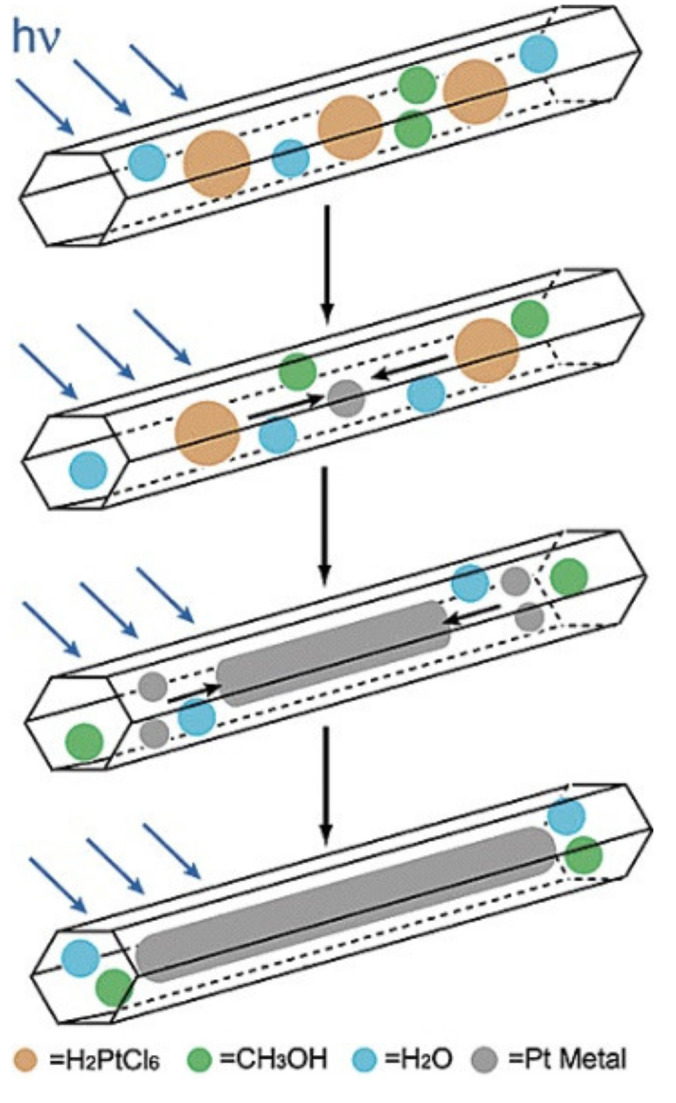
The mechanism of nanowires formation in HMM-1 channels under the effect of UV irradiation, proposed by Fukuoka et al. Reprinted with permission from Reference [106]. Copyright (2004) American Chemical Society.

Fukuoka et al. have explained the mechanism of formation of Pt nanowires and nanoparticles (Figure 13), on the basis of TEM images, X-ray absorption fine structure spectroscopy (XAFS) and the material response to changes in the time exposition to UV radiation. According to their proposition, PtCl_6_^2−^ ions were reduced on the surfaces of Pt nanoparticles formed initially in the mesopores as a result of photoreduction. Most probably water and methanol acted as solvents in the HMM-1 pores, so Pt^4+^ ions were able to migrate in the liquid phase and reach the surface of the earlier formed small Pt nanoparticles. Then the Pt^4+^ ions were reduced by the solvated electrons and free radicals produced under irradiation. Exposition to gas hydrogen resulted in formation of nanoparticles or short nanowires as the reduction was faster than migration of platinum ions [105,106,107].

The Pt nanowires can be separated from the HMM-1 pores through dissolution of this silica material in a diluted solution of HF [106], filtration and washing with ethanol. According to the TEM images, the separated Pt nanowires maintained their original structure and did not undergo fusion. They were stable solid at 5 °C, while as suspension in ethanol they aggregated forming large molecules. Addition of the stabilizing ligands, for example, ODTMACl or P(C_6_H_5_)_3_, to the HF solution prevented the Pt nanowires aggregation. The Pt nanowires modified with ligands have been found much promising for production of highly-ordered nanostructures [106]. Fukuoka et al. [100] have also synthesized palladium nanowires and nanoparticles in 1D channels of HMM-1 and the obtained Pd-HMM-1 composites were used in CO oxidation. To obtain such composites, HMM-1 was impregnated with as-prepared solution of H_2_PdCl_4_ and exposed to a stream of oxygen or nitrogen, at 200 °C. In order to obtain the active phase (palladium nanowires or nanoparticles), the materials were subjected to reduction in a hydrogen atmosphere at 200 °C or photoreduction using water and methanol and UV radiation then exposition to gas hydrogen. According to the authors, palladium nanowires were formed as a result of photoreduction followed by reduction with H_2_, while Pd nanoparticles were obtained as a result of a one stage reduction—exposition to H_2_. In the test reaction of CO oxidation the surfaces of palladium nanowires were found to be more active than palladium nanoparticles as indicated by higher TOF (turnover frequency) values [100].

Metal nanowires can be formed in HMM-1 pores also as a result of reduction of a metal precursor with steam-saturated hydrogen (wet H_2_-reduction), which has been shown by Fukuoka et al. [101]. They have also studied the synthesis of platinum and gold nanowires and nanoparticles in HMM-1 channels. As follows from their results, reduction of the metal precursors with steam-saturated hydrogen was conducive to formation of nanowires as steam added to hydrogen facilitated migration of Pt ions in 1D HMM-1 channels, leading to the growth of nanowires. The reduction with gas hydrogen with no steam resulted in formation of nanoparticles [101]. HMM-1 containing platinum nanoparticles have been applied as catalysts for preferential oxidation of carbon oxide (PROX reaction) [108,109,110], however they showed low catalytic activity. In contrast, the Folded Sheet Materials (FSM-16) with identical structure of pores containing Pt nanoparticles, showed high activity and selectivity in the same reaction. The above observation implied the conclusion that the mesoporous structure of silica and pore hydrophilicity had a significant influence on catalytic activity [111].

The materials of 3D hexagonal structure, HMM-2, have been also applied as templates for synthesis of metal nanoparticles. Fukuoka et al. [112] have synthesized gold and platinum nanoparticles in the 3D pore structure of HMM-2. This HMM-2 sample was obtained in basic conditions with the use of ODTMACl and NaOH dissolved in water, then BTME was added and the reaction mixture was stirred at room temperature for 24 h. The suspension obtained was filtered off, dried and extracted with a solution of HCl in ethanol to remove the surfactant. The material obtained was used as a matrix for the synthesis of gold or platinum nanoparticles from the precursors HAuCl_4_ or H_2_PtCl_6_·6H_2_O, respectively. Impregnation with gold precursor took place in basic conditions at pH of 11.7 achieved by addition of NaOH. The sample impregnated with platinum precursor was subjected either by photoreduction or by reduction with gas hydrogen, while the sample impregnated with gold precursor was subjected only to reduction with gas hydrogen. It has been shown that nanoparticles of platinum and gold were successfully synthesized in HMM-2 pores. The gold nanoparticles were homogeneously distributed in HMM-2, whose structure was preserved after formation of gold nanoparticles of the average size of 3.2 ± 0.5 nm. The average size of platinum nanoparticles isolated from HMM-2 with the use of HF solution containing triphenylphosphine (PPh_3_), was 2.5 ± 0.8 nm [112].

Bore et al. [113] and Gabaldon et al. [114] have studied the effects of pore size and mesoporous structure of silicas on the control of thermal sintering of gold nanoparticles. They synthesized mesoporous silicas of different structures: MCM-41 and SBA-15 of 2D hexagonal structure, SBA-12 and HMM-2 of 3D hexagonal structure and SBA-11 of cubic structure. Prior to the impregnation with gold precursor, the surface of silica was grafted with amino groups from (3-Aminopropyl)trimethoxysilane, which facilitated the precursor adsorption. The silicas modified with amino groups were added to a water solution of HAuCl_4_ of pH 7 (controlled with NaOH). After the impregnation the materials were reduced with gas hydrogen at 200 °C for 2 h. The gold nanoparticles were measured by the HAADF-STEM method to show that their size is greater than the diameters of the pores of the silica materials studied except for SBA-15 of relatively great pores. Thus, it has been concluded that Au nanoparticles destroy the pore walls and grow continuously in the process of reduction, which suggests that Ostwald maturation was the main mechanism in the gold nanoparticles sintering. The obtained catalysts were tested in the CO oxidation taking place from room temperature to 400 °C. Although the gold particles were large enough to block the pores, the catalysts tested were active in the reaction. It means that the thin silica walls ensured easy access to the gas phase. The gold catalyst based on HMM-2 showed the highest catalytic activity in the reaction run at 100 °C, from among the other gold catalysts tested (SBA-15, SBA-12, SBA-11, MCM-41). It should be added that after the reaction the diameters of gold nanoparticles were greater than prior to the reaction and after the reduction [113,114].

## 5. Comparison of SBA and HMM Materials

Table 1 shows a comparison of type SBA-1, SBA-2, HMM-1 and HMM-2 materials, taking into account the main differences between their special features.

## 6. Application of Mesoporous Silica Materials—Summary

Nanoporous silica materials, due to their physicochemical properties, show a number of applications [115,116,117]. Table 2 summarizes potential applications of SBA-1, SBA-2, HMM-1 and HMM-2 materials.

## 7. Summary

The paper presents the syntheses, physicochemical characterization and applications of SBA-1, SBA-2, HMM-1 and HMM-2 silicas based on literature peruse. SBA-1 silicas have been initially synthesized in acidic conditions with the use of cationic surfactants. However, the acidic environment during the synthesis limited the introduction of metal ions to the skeleton and on the surface of SBA-1. Due to the high potential of SBA-1-type materials to be used in processes such as adsorption and catalysis, the scientists have improved the procedures for their synthesis. By modifying the synthesis procedure and the reagents used, it is possible to influence, among others, the specific surface area and the shape and volume of the final material pores. The syntheses were carried out with the use of polyelectrolyte-surfactant complex as a template in alkaline environment, which turned out to be a better alternative to the previously carried out syntheses (acidic environment, cationic surfactant). The proposed methods of syntheses of SBA-1 modified with metal ions have been proved to lead to catalysts that can be applied in a number of reactions, including: oxidation, condensation, acetalization, epoxidation, isomerization of different organic compounds or dehydrogenation of alkanes. The silicas modified with functional groups (amino, carboxyl) have found application in adsorption of oxyanions, dyes or to immobilization of enzymes.

This literature review shows that SBA-1 materials have very high potential for adsorption and catalyst applications. Currently, these materials are not as appreciated as other SBA materials (SBA-15, SBA-16), however, their great advantage is the possibility to control their structure and texture parameters already at the synthesis stage. In addition, the possibility of modifying their structure with metal ions and organic functional groups opens the door to designing catalysts adapted to specific applications.

Replacement of a gemini type surfactant with a polyelectrolyte-surfactant complex as a template in the synthesis of SBA-2 has been promising for the synthesis of metal ions modified silicas. So far, these materials have been modified with titanium ions, so that the obtained materials served as catalysts for oxidizing desulfurization of Diesel oil. It would be worthwhile to focus on the introduction of other metal ions into the structure and/or the surface of SBA-2, which would probably increase their potential for use in many industries.

Hybrid mesoporous materials (HMM) belonging to PMOs, have been applied first of all for the syntheses of nanowires and metal nanoparticles inside the HMM pores. HMM-1 silicas modified with rhodium or platinum and subjected to supercritical carbon(IV) oxide, have proved to be catalytically active for hydrogenation of light alkanes, while when modified with sulfonic groups—for hydrolysis of saccharides in which they were much more active than the commercial catalysts.

PMOs materials, due to their multitude of synthesis variants and the presence of organic functional groups inside the pore walls, can be promising candidates for many applications, including chromatography, nanoelectronics, adsorption, catalysis, as well as can be used for the release of active substances.

## Figures and Tables

**Table 1:** Comparison of SBA and HMM materials.

	SBA-1	SBA-2	HMM-1	HMM-2
**First synthesis**	1994	1995	1999	1999
**Type of material**	Mesoporous Silica Materials		Periodic Mesoporous Organosilicas (PMOs)	
**Type of structure**	3-dimentional (3D) cubic	3-dimentional (3D) mixed hexagonal and cubic	2-dimentional (2D) hexagonal	3-dimentional (3D) hexagonal
**Symmetry**	Pm$\bar{3}$n	P6_3_/mmc	p6mm	P6_3_/mmc
**Surface area (m^2^/g)**	1200–1450	~600	~1000	~1200
**Pore size (nm)**	2.1–2.6	3.5	3.1	2.7
**Position of main X-ray reflections (2θ)**	2°–3°	2°–7°	1.8°–4°	2°–4°
**Crystallographic planes (hkl)**	(200), (210), (211)	(100), (002), (101), (110), (103), (112), (211)	(100), (110), (200)	(100), (002), (101), (110), (103), (112)

**Table 2:** Potential use of the SBA and HMM-type materials.

Type of material	Metal/Organic Groups	Application	Ref.
**Synthesis in Acidic Conditions**			
SBA-1	Ti	Oxidation of styrene with hydrogen peroxide	[43]
SBA-1	Fe		
SBA-1	Mo	Partial oxidation of methane with oxygen	[49]
SBA-1	Al	Isomerization of *n*-decane	[51]
SBA-1	Al	Synthesis of 7-methoxy-4-methylcoumarin	[52]
SBA-1	Al Al and Mg	Acetalization of heptanal	[53]
SBA-1	Ti	Epoxidation of styrene to styrene oxide	[54]
SBA-1	Cr	Dehydrogenation of ethane with the use of CO_2_	[56]
		Dehydrogenation of propane with the use of CO_2_	[57]
SBA-1	Ga	*tert*-butylation of phenol	[25]
		Alkylation of naphthalene with propylene	[58]
SBA-1	Mn	Oxidation of ethylbenzene with the use of TBHP	[59]
SBA-1	alkali metals Li, Na, K, Rb, Cs	Knoevenagel condensation between benzaldehyde or benzylacetone and ethyl cyanoacetate	[60]
SBA-1	amino groups	Adsorption of oxyanions (chromates and arsenates)	[62]
SBA-1	Hoveyd-Grubbs catalyst	Olefin metathesis catalyst	[67]
SBA-1	unmodified	For obtaining highly-ordered carbon materials	[68]
**Synthesis in Basic Conditions**			
SBA-1	Al	Alkylation of 2, 4-Di-*tert*-butylphenol with cinnamyl alcohol	[69,70]
		Alkylation of toluene with benzyl alcohol	[72]
SBA-1	Ti	Oxidation of 2, 3, 6-trimethylphenol	[73]
SBA-1	unmodified	Immobilization of lysozyme	[74]
SBA-1	carboxyl groups	Immobilization of papain	[76]
SBA-1	carboxyl and amino groups	Adsorption of toxic anionic or cationic dyes	[77]
SBA-2	thiol groups	Esterification of glycerol with oleic acid (very low activity)	[92]
SBA-2	sulfonic groups	Esterification of glycerol with oleic or lauric acid (very low activity)	[84]
SBA-2	Ti	Oxidizing desulfurization of Diesel oil	[79]
SBA-2	unmodified	Adsorbent of volatile organic compounds (adsorbent for separation of a mixture of benzene/cyclohexene)	[94]
HMM-1	Rh or Pt and Rh	Hydrogenation of *n*-butane	[98]
HMM-1	sulfonic groups	Hydrolysis of saccharose of starch	[103]
HMM-1	unmodified	Matrices for syntheses of nanowires and metal nanoparticles (bimetallic Pt-Rh, Pt-Pd as well as monometallic Pt or Rh)	[104]
HMM-1	Pd nanowires or nanoparticles	CO oxidation	[100]
HMM-2	unmodified	Matrices for syntheses of nanowires and metal nanoparticles (Au, Pt)	[112]
HMM-2	Au nanoparticles	CO oxidation	[113,114]

## Abbreviations

APTMS	(3-Aminopropyl)trimethoxysilane
BJH	Barrett- Joyner-Halenda method
BTME	1, 2-Bis(trimethoxysilyl)ethane
BTMB	1, 4-Bis(triethoxysilyl)benzene
C_16_TAB	hexadecyltrimethylammonium bromide
C_16_TEAB	hexadecyltriethylammonium bromide
C_16_TPAB	hexadecyltripropylammonium bromide
C_18_TMACl = ODTMACl	octadecyltrimethylammonium chloride
CES	carboxyethyl silanetriol sodium salt
CPC	hexadecylpyridinium chloride
FDU	group of mesoporous materials named after Fudan University
FSM	group of mesoporous materials named after Folded Sheet Materials
HAADF- STEM	high-angle annular dark-field imaging scanning transmission microscopy
HMM	group of mesoporous materials named after Hybrid Mesoporous Materials
HRTEM	high-resolution transmission electron microscopy
IEP	isoelectric point
IUPAC	International Union of Pure and Applied Chemistry
KIT	group of mesoporous materials named after Korean Advanced Institute of Science and Technology
MCM	group of mesoporous materials named after Mobile Composition of Matter
MPTMS	(3-Mercaptopropyl)trimethoxysilane
MSU	group of mesoporous materials named after Michigan State University
PAA	poly(acrylic acid)
PMOs	Periodic Mesoporous Silicas
SBA	group of mesoporous materials named after Santa Barbara University
SEM	scanning electron microscopy
scCO_2_	supercritical carbon(IV) oxide
TBHP	*tert*-Butyl hydroperoxide
TEM	transmission electron microscopy
TEOS	tetraethyl orthosilicate (tetraethoxysilane)
TGA	thermogravimetric analysis
THF	tetrahydrofuran
TMB	1,3,5-trimethylbenzene
TMOS	Tetramethyl orthosilicate (tetramethoxysilane)
TOF	turnover frequency
TPOS	tetrapropyl orthosilicate (tetrapropoxysilane)
UV-VIS	ultraviolet-visible spectroscopy
XAFS	X-ray absorption fine structure spectroscopy
XRD	X-ray diffraction
